# Wireless, Deep‐Seeing Smart Pill: A NIR‐II Fluorescence Imaging Capsule Endoscope for Gastrointestinal Cancer

**DOI:** 10.1002/advs.75532

**Published:** 2026-05-12

**Authors:** Weicheng Wang, Jinlei Jiang, Zhengting Wang, Shuqi Liu, Wei Wang, Xi Chen, Qirui Zhao, Xinyuan Cui, Cheng Zhou, Shengsheng Cui, Daxiang Cui

**Affiliations:** ^1^ School of Sensing Science and Engineering School of Electronic Information and Electrical Engineering Shanghai Jiao Tong University Shanghai P. R. China; ^2^ Department of Gastroenterology Ruijin Hospital Shanghai Jiaotong University School of Medicine Shanghai P. R. China; ^3^ Global Institute of Future Technology Shanghai Jiao Tong University Shanghai P. R. China; ^4^ Medical and Engineering Cross Research Institute the First Affiliated Hospital of Henan University Kaifeng P. R. China; ^5^ Department of Radiology Ruijin Hospital Shanghai Jiao Tong University School of Medicine Shanghai P. R. China

**Keywords:** capsule endoscopy, dual‐mode imaging, early gastrointestinal cancer, fluorescent nanoprobe, near‐infrared‐II fluorescent

## Abstract

Gastrointestinal cancer detection technologies are evolving; capsule endoscopy (CE) overcomes the invasiveness of tethered endoscopes. However, current CEs exhibit limited penetration depth and low SNR due to strong photon scattering, absorption, and autofluorescence in biological tissues, potentially resulting in missed deep or small lesions. This study reports a near‐infrared‐II fluorescent imaging capsule endoscope (NIR‐II FICE), a “smart pill” designed for early gastrointestinal cancer diagnosis. NIR‐II FICE can detect NIR‐II‐specific fluorescence signals above 900 nm from lesional tissues using the NIR‐II fluorescent nanoprobe (FNP). It possesses the advantages of high sensitivity, high resolution, strong tissue penetration ability, high SNR, and high‐contrast imaging. Experiments show NIR‑II FICE can detect FNP at concentrations as low as 5 µg mL^−1^ and image large‑scale irregular regions and minute areas down to 100 µm. In ex vivo experiments on porcine gastric tissue, the penetration depth achieved 4 mm, with SNRs exceeding 13 dB and contrast values all greater than 0.5. In vivo experiments enabled targeted imaging of lesions in nude mice. Furthermore, a wireless‐powered, magnetically controlled device ensures continuous energy and precise motion for NIR‑II FICE. To our knowledge, this is the first CE platform for NIR‐II fluorescence imaging, advancing non‐invasive gastrointestinal disease diagnosis/treatment and clinical research.

## Graphical abstract

A novel near‐infrared‐II fluorescent imaging capsule endoscope that detects specific fluorescence signals above 900 nm enables highly sensitive, targeted imaging of gastrointestinal cancer tissue. This wirelessly powered and magnetically controlled “smart pill” achieves high sensitivity and deep tissue penetration, allowing precise, non‐invasive localization of minute lesions and offering a transformative platform for clinical disease detection.

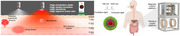

## 1 Introduction

Gastrointestinal cancer presents a considerable threat to human health, accounting for roughly one‐quarter of the global cancer incidence and up to one‐third of cancer‐related deaths [1]. In the early stage, tumors are generally small and non‐metastatic. Treating tumors at this stage can remarkably enhance patients’ 5‐year survival rates. However, this advantage depends on the tumor being detected at a potentially curable stage. Thus, early and precise diagnosis is crucial for improving the survival outcomes and prognosis of patients with gastrointestinal cancer [2, 3, 4].

Currently, tethered endoscopy remains the gold standard for gastrointestinal cancer detection. However, it is accompanied by several limitations. Specifically, the invasiveness of the procedure may result in patient discomfort and potentially cause traumatic damage to the gastrointestinal tract [5, 6]. The advent of CE has ushered in a new era of painless and non‐invasive examination of the digestive tract, and it has experienced rapid development and innovation [7, 8]. Commercial CE systems predominantly employ white light imaging (WLI) technology. For example, companies such as Medtronic, Olympus, Jinshan, CapsoVision, Intromedic, Motilis, and Ovesco have developed WLI CEs for the examination of different segments of the digestive tract, including the small intestine [9, 10, 11], colon [12], and esophagus [13]. These WLI CEs allow for non‐invasive and sedation‐free detection of the entire gastrointestinal tract. However, the limited penetration depth of white light impedes the clear visualization of submucosal and deeper tissue lesions [8]. Additionally, the limitations of wireless data transmission can result in inadequate resolution, which may potentially compromise the depiction of early‐stage micro‐lesions and thus increase the risk of missed diagnoses. Consequently, over the past few decades, numerous researchers have utilized CE as a research platform to develop various types of CEs, with the aim of enhancing penetration depth, improving resolution, and optimizing imaging quality. Ultrasound imaging technology has sparked fierce competition among numerous leading research teams globally due to its high penetration capability. These teams are actively engaged in developing CEs integrated with high‐frequency miniature ultrasound (µUS) to obtain high‐resolution images [14, 15, 16]. Although certain researchers have manufactured capsules of the requisite dimensions, the development of ultrasound‐based CE is still in its infancy, and many technical challenges still need to be tackled in the fabrication and integration of appropriate ultrasonic transducers [14, 17]. Optical coherence tomography (OCT), characterized by its micrometer‐scale resolution, has exhibited outstanding performance in the diagnosis and evaluation of gastrointestinal mucosal injuries, Barrett's esophagus, early gastrointestinal cancers, and inflammatory bowel disease [18, 19, 20]. Based on OCT technology, tethered CEs integrated with OCT have been developed for gastrointestinal tract examination [21, 22, 23, 24, 25], which can achieve high‐resolution imaging. However, challenges such as power supply and scanning under peristaltic motion still need to be tackled to implement a truly practical CE [8]. Narrow‐band imaging CE capitalizes on the disparities in the absorption and reflection characteristics of biological tissues at different wavelengths to enhance imaging contrast. This approach effectively improves the contrast of mucosal and microvascular visualization [26, 27], subsequently enhancing the image quality and the lesion detection rates. However, short‐wavelength light experiences intense scattering, and it has a limited penetration depth in tissue. As a result, when imaging deep tissue structures or thick mucosal layers, the clarity is inadequate, which restricts the ability to evaluate the depth and extent of lesions. Additionally, in this spectral region, tissue autofluorescence is relatively prominent, leading to an increase in background noise and a reduction in SNR.

With the advancements in fluorescence imaging (FI) technology, fluorescent CE has increasingly emerged as a crucial tool in the diagnosis of digestive tract diseases. By combining the non‐invasiveness of CE with the high sensitivity of FI, this approach provides novel perspectives for the detection of early‐stage lesions. Currently, the FI techniques employed in clinical practice mainly consist of autofluorescence imaging and near‐infrared region I (NIR‐I) FI. Autofluorescence imaging makes use of violet light to excite endogenous fluorophores for visualization, which allows for the differentiation of fluorescence signals between tumors and the surrounding normal tissues without the requirement of exogenous labeling. However, the existing autofluorescence CE systems predominantly depend on single‑photon avalanche diode (SPAD) [28, 29] or SPAD arrays [30] to obtain fluorescent signals through photon counting, instead of realizing true 2D imaging. As a result, the spatial resolution is considerably inadequate for clinical applications [31]. Furthermore, autofluorescence imaging frequently uses excitation sources with a wavelength of 420 nm, which has poor tissue penetration and is generally limited to the superficial mucosal layer. Meanwhile, signal interference from non‑target regions occurs because various biological tissues produce autofluorescence within this wavelength range. An NIR‑I FICE operates by using excitation light at 680 or 740 nm to excite the indocyanine green fluorescent dye. This dye emits characteristic fluorescence signals within the first NIR window (700–900 nm), which enables the identification of pathological tissues [32, 33], and helps overcome false‑positive results caused by background autofluorescence in autofluorescence imaging. However, current NIR‑I FICE systems still need improvements in the integration of light sources and optical filters and in tissue penetration depth. Compared with the NIR‐I window, the NIR‐II region exhibits diminished photon scattering and absorption. Consequently, imaging utilizing light within the NIR‐II wavelength range of 900–1700 nm allows for deeper tissue penetration, higher spatial resolution, and enhanced overall image quality [34, 35]. Although NIR‐II FI has shown substantial promise in biomedical research, the majority of current imaging systems are founded on bulky, box‐like designs and are primarily restricted to open surgical field applications or ex vivo benchtop imaging setups [36, 37, 38, 39]. For in vivo applications, particularly in the detection of gastrointestinal cancers, these systems still encounter several limitations such as difficulties in system integration and an insufficient power supply. Consequently, the development of an NIR‐II FICE capable of performing intracorporeal examinations within the gastrointestinal tract has become an urgent need in the field of medical imaging.

**FIGURE 1:**
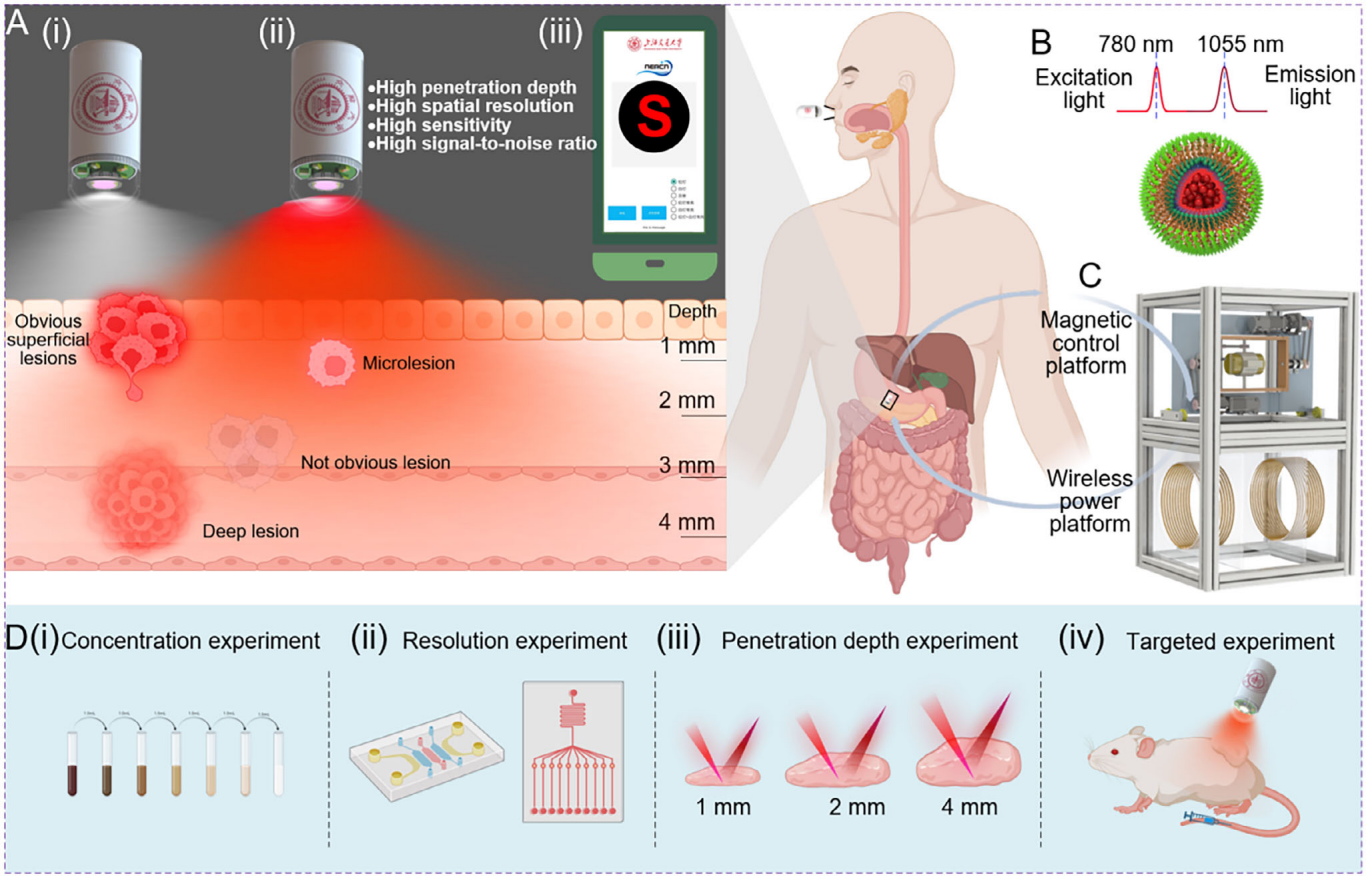
Schematic of NIR‑II FICE detection system. (A) Dual‑mode imaging capability of NIR‑II FICE and its associated advantages. (B) NIR‑II FNP. (C) Integrated wireless power supply and magnetic control module. (D) Experimental demonstrations of NIR‑II FICE: (i) assay of minimum detectable concentration, (ii) evaluation of spatial resolution, (iii) assessment of depth detection, and (iv) verification of specific targeting.

In response to the limitations of existing endoscopic technologies and capitalizing on the advantages of NIR‑II FI, an “intelligent pill” named NIR‑II FICE was developed for the early diagnosis of gastrointestinal cancer. This intelligent pill integrates a light‑source module, an image acquisition module, a microcontroller module, a wireless image‑transmission module, a wireless power‑reception module, and an attitude‑control module. All these functional units are incorporated into a capsule shell with overall dimensions of 12 mm × 37 mm. The proposed “smart pill” is characterized by dual‑mode imaging and detection capability. In WLI mode, it can provide illumination and perform positional localization (Figure 1A‐i). In FI mode, it can capture NIR‑II fluorescence (900–1200 nm) emitted from pathological tissues specifically targeted by NIR‑II FNP (Figure 1B), as depicted in Figure 1A‐ii, and then transmit the acquired signal to an external receiving device (Figure 1A‐iii). Furthermore, an integrated platform that combines wireless power transfer and magnetic actuation was established (Figure 1C). As a proof of concept, NIR‐II FICE was employed to carry out relevant experiments on a fluorescent nanoprobe solution (Figure 1D‐i), an irregular chip Figure 1D‐ii), an ex vivo pig stomach (Figure 1D‐iii), and a live mouse (Figure 1D‐iv). NIR‑II FICE enables the detection of FNP solutions with concentrations as low as 5 µg mL^−1^. It can effectively examine large irregularly shaped regions and miniature areas with a size down to 100 µm. Moreover, it can penetrate ex vivo porcine gastric tissue up to a depth of 4 mm and conduct targeted imaging of pathological tissues in living subjects. It demonstrates high sensitivity, high spatial resolution, strong tissue penetration ability, increased SNR, and excellent image contrast for detection applications. In addition, the integrated device combining wireless power supply and magnetic control can continuously supply energy to NIR‐II FICE and realize motion control. To the best of the authors’ knowledge, this work represents the first implementation of an NIR‑II FICE system, which provides high‑quality imaging modality for the early detection of gastrointestinal cancers and holds promise as a pivotal tool in clinical diagnosis.

## 2 Results and Discussion

### 2.1 Assembly and Testing of NIR‐II FICE

**FIGURE 2:**
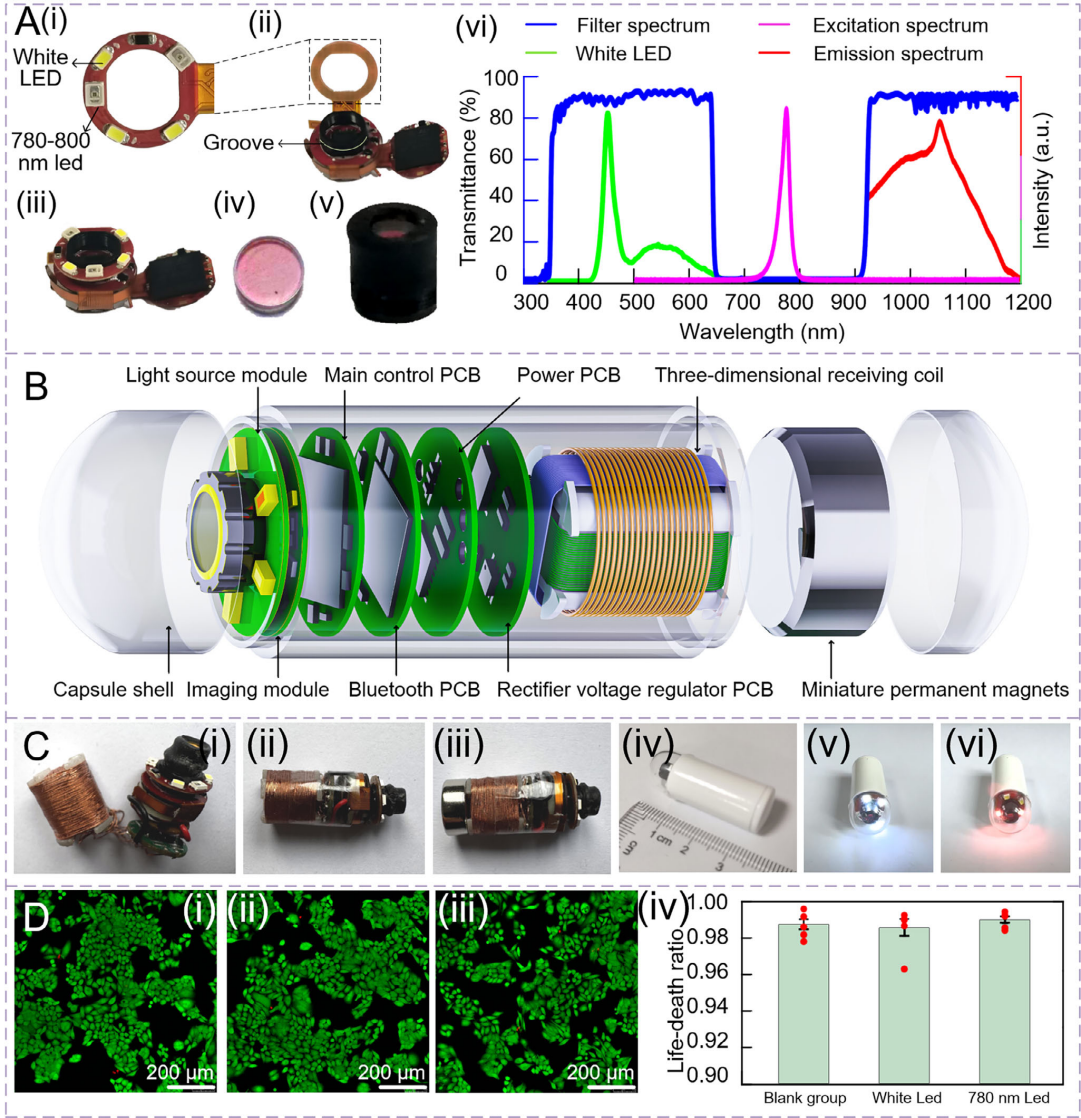
NIR‐II FICE assembly and testing. (A‐i) light source module of NIR‐II FICE; (ii) separately designed light source module and image acquisition module; (iii) light source module fixed within the lens mount groove; (iv) notch filter; (v) encapsulated filter and lens assembly; and (vi) spectra of the light source, filter, and FNP. (B) Schematic depicting the NIR‐II FICE structure. (C) Assembly results of NIR‐II FICE. (D) (i) Confocal live/dead cell images of the control group, (ii) confocal live/dead cell images obtained under the excitation of NIR light source, (iii) confocal live/dead cell images obtained under the excitation of a white light source, and (iv) live/dead ratios from six experiments.

In this study, a custom‐designed NIR‐II FICE light source module was developed, which integrates two types of LEDs: an NIR LED with a central wavelength of 780 nm (2016 package) and a broadband white‐light LED (0603 package). The LEDs were evenly distributed and soldered onto a rigid annular printed circuit board (PCB) with inner and outer diameters of 6.6 and 10.2 mm, respectively (Figure 2A‐i). This design satisfies the optical performance requirements while achieving a balance between the miniaturization and spatial layout flexibility of the device. White‐light LED provides uniform illumination, enhancing mucosal morphology visualization and facilitating real‐time navigation and macroscopic screening. The NIR‐II fluorescence mode utilizes excitation light with a center wavelength of 780 nm, which exhibits low absorption and scattering characteristics within biological tissues. This confers superior penetration depth and enhanced spatial resolution, specifically enabling the detection of molecular fluorescence signals from deep‐seated lesions. The system achieved complementary fusion of dual‐modal imaging, thus facilitating a comprehensive evaluation of tumor location, morphology, and infiltration depth. Additionally, both LEDs have an emission angle of up to 120°, which enables wide‐field uniform illumination. This wide‐field illumination ensures complete coverage of the gastrointestinal tract wall without blind spots, thereby enhancing the accuracy and comprehensiveness of diagnosis. However, NIR excitation light with high penetration capability can easily enter the complementary metal‐oxide‐semiconductor (CMOS) sensor, thus introducing considerable background noise. A multi‐level optical path management strategy was adopted to suppress this interference. First, the junction between the lens base and the image sensor PCB was hermetically sealed with opaque UV‐curable adhesive. Second, from the perspective of structural design, the PCB of the light source module and the PCB of the image acquisition module carrying the CMOS sensor were separately designed and fabricated to physically isolate the excitation light path (Figure 2A‐ii). Simultaneously, a stepped groove structure was designed on the lens mount. Once the lens mount was firmly fixed, the light‐source ring PCB was embedded into the mount groove, which was positioned 1.75 mm above the imaging surface of the sensor chip. These strategies effectively block the propagation path of the excitation light through the mount gaps to the CMOS sensor (Figure 2A‐iii). Third, at the optical level, a notch filter was integrated at the front end of the lens to further prevent the excitation light and the reflected light from entering the CMOS sensor through the lens (Figure 2A‐iv). The analysis of the emission spectra of the NIR and white‐light sources [pink and green curves in Figure 2A‐vi, the transmission spectrum of the filter [blue curve in Figure 2A‐vi], and the emission spectrum of FNP [red curve in Figure 2A‐vi] showed that this filter can efficiently transmit visible light in the 350–650 nm range and NIR‐II fluorescence emission in the 900–1200 nm range while effectively blocking the excitation light from the NIR source. Finally, an opaque heat‐shrink tube was employed to wrap and seal the bonding area between the filter and the micro‐lens to further prevent the excitation light and reflected light from seeping into the lens cavity through the bonding gap between the filter and the lens, thereby achieving full‐path optical isolation (Figure 2A‐v). Through the synergistic effect of the aforementioned hardware structural optimization and optical filtering design, the system can effectively suppress the background interference caused by the excitation light source, thereby ensuring a high SNR and high‐quality imaging in NIR‐II FI.

The light source module, image acquisition module, microcontroller module, wireless image transmission module, and power supply module were subsequently integrated onto a flexible‐rigid PCB (Figure S1). The 3D receiving coil and the rectifier‐stabilizer PCB with a diameter of 10.1 mm were then connected to the flexible‐rigid PCB. Subsequently, they were encapsulated together with a miniature permanent magnet inside the capsule shell. The schematic of the assembly process is shown in Figure S2. Figure 2B presents an exploded schematic of NIR‐II FICE, and Figure 2Ci–iv shows the key assembly results of NIR‐II FICE. The assembled NIR‐II FICE can operate in WLI mode (Figure 2C‐v) and NIR‐II FI mode (Figure 2C‐vi). To investigate the interactions between NIR‐II FICE and the gastrointestinal environment, the FICEs were immersed in simulated gastric fluid, deionized water, and a sodium bicarbonate solution for 30 min each (Figure S3A–C). The results showed that the pH of these solutions remained essentially constant, with fluctuations confined within ±0.03 (Figure S3D–F). This stability indicates that the NIR‐II FICE device does not exert a significant impact on the local gastrointestinal pH during standard operation. Furthermore, optical imaging was performed following the 30‐minute immersion. As illustrated in Figure S3G–I, the CEs maintained high‐clarity imaging, demonstrating that the capsule shell effectively safeguards the internal electronic components and ensures robust optical performance. Subsequently, the viability of cells was evaluated by utilizing the two light sources of NIR‐II FICE. The confocal live/dead stained images are shown in Figure 2Di–iii, which correspond to the control group, cells excited by the NIR light source, and cells excited by the white light source, respectively. Six repeated experiments were conducted to eliminate randomness in the experiment (Figure S4). Then, the live‐to‐dead ratios were calculated using ImageJ software. As shown in Figure 2D‐iv, the live‐to‐dead ratios of all six experiments exceeded 95%, indicating that the prototype light source of NIR‐II FICE exhibited excellent biocompatibility.

### 2.2 Synthesis and Characterization of NIR‐II FNP

**FIGURE 3:**
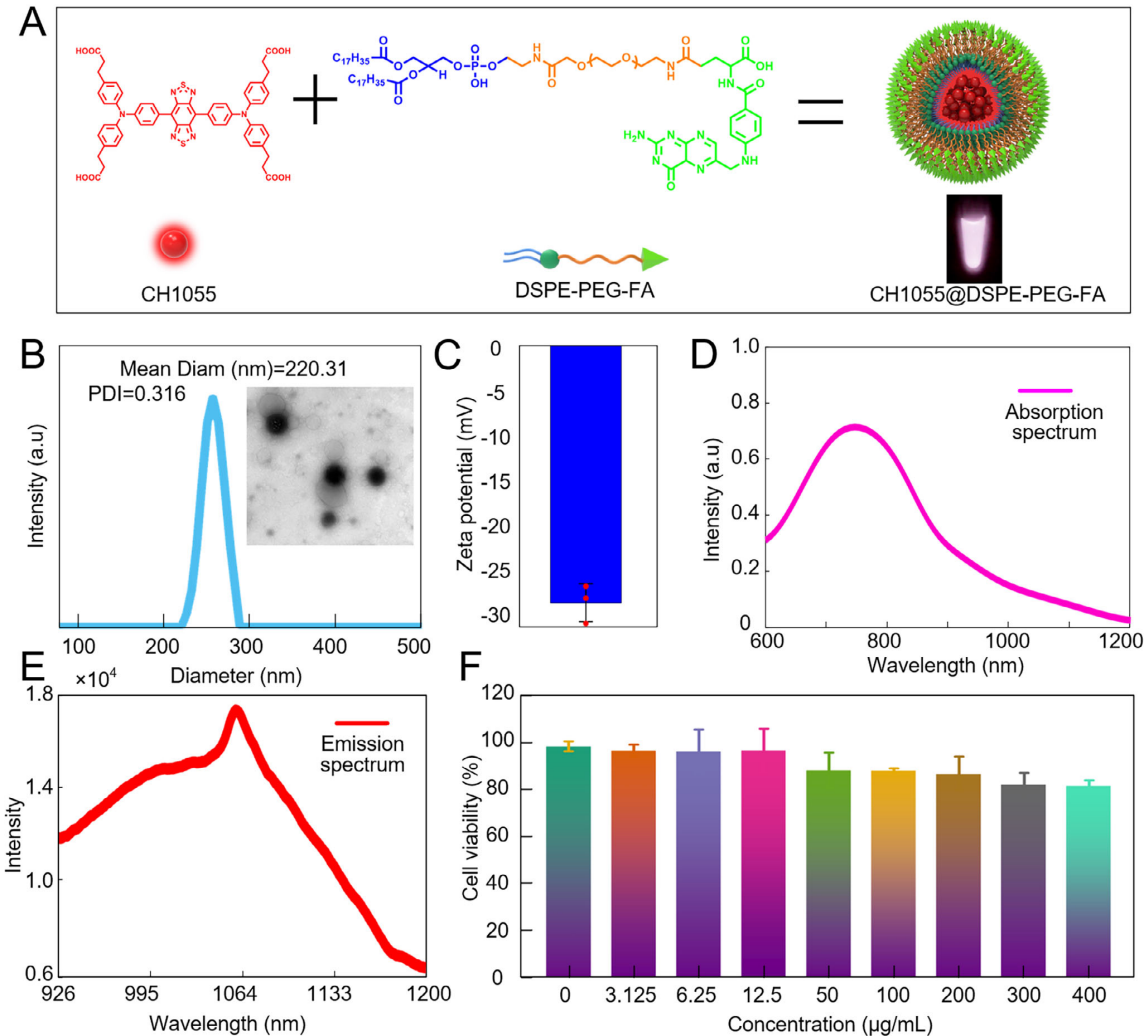
Synthesis and characterization of NIR‐II FNP. (A) Composition of NIR‐II FNP. (B) Transmission electron microscopy (TEM) image and particle size distribution of NIR‐II FNP. (C) Zeta potential of NIR‐II FNP. (D) Absorption spectrum of NIR‐II FNP. (E) Emission spectrum of NIR‐II FNP. (F) Cell viability assay of NIR‐II FNP.

CH1055 fluorescent materials exhibit remarkable advantages owing to their well‐defined and tunable chemical structures, along with favorable metabolic properties. These characteristics endow them with extensive application prospects in the field of biomedical imaging. Consequently, the present study was designed on the basis of CH1055 fluorescent materials. CH1055 was loaded into nanoparticles composed of DSPE‐PEG‐FA, which is an amphiphilic diblock copolymer commonly used in drug targeted delivery system, using ultrasonic nanoprecipitation method (Figure 3A).

Subsequently, TEM imaging was carried out on the synthesized NIR‐II FNP. The results showed spherical nanoparticles, as shown in the upper‐right image of Figure 3B. The Dynamic light scattering (DLS) results revealed that the average hydrodynamic diameter and polydispersity index (PDI) of CH1055@DSPE‐PEG‐FA were approximately 220.31 nm and 0.316, respectively, with a zeta potential of roughly –25 mV (Figure 3C). Continuous monitoring over a three‐week period (Figure S5) confirmed the particle size uniformity, superior dispersibility, and exceptional colloidal stability of the CH1055@DSPE‐PEG‐FA nanoparticles. The absolute quantum yield of the NIR‐II FNP was determined to be approximately 0.5%. The absorbance testing of NIR‐II FNP yielded the absorption spectrum shown in Figure 3D, which exhibits an absorption peak around 780 nm. Water shows low absorption and scattering within this wavelength range. This unique “optical transparency window” characteristic enables NIR‐II FICE to penetrate more deeply into biological tissues. The emission spectrum of FNP, as depicted in Figure 3E, exhibited an emission peak at around 1055 nm. The full width at half maximum of this emission peak was roughly 100 nm, and the intensity of the fluorescence signal declined to almost zero in the vicinity of 1200 nm. This broad‐bandwidth emission spectrum serves to enhance the photon flux and signal accumulation, encompasses the CMOS response window within the NIR‐II region, thereby improving the overall signal capture efficiency. Moreover, within the NIR‐II wavelength band, the autofluorescence intensity of biological tissues was notably reduced. This reduction can effectively minimize the interference caused by tissue autofluorescence, enhance the SNR and the clarity of imaging, and facilitate the long‐distance travel of photons within biological tissues, thus achieving enhanced imaging depth. Meanwhile, the reduction in light scattering contributes to the enhancement of spatial resolution in imaging, which allows for clear observation of the structures and details in deep tissues. To assess the fluorescence stability, the emission spectra were recorded after continuous exposure to the excitation light source for 5, 10, 15, 20, 25, and 30 min. Integration of the spectral area within the 900–1200 nm range yielded the results shown in Figure S6. The fluctuations in integrated fluorescence intensity remained within 5%, demonstrating the superior photostability of the NIR‐II FNP. Subsequently, CCK‐8 cell viability assays were carried out using the prepared NIR‐II FNP, and the experiments were repeated three times. The cytotoxicity of CH1055@DSPE‐PEG‐FA on gastric epithelial cells (GES‐1) cells was detected at a concentration gradient (Figure 3F). The results showed that a high concentration of CH1055@DSPE‐PEG‐FA (400 µg mL^−1^) still exhibited good biocompatibility and safety for normal cells.

### 2.3 NIR‐II FICE Imaging Experiments

#### 2.3.1 NIR‐II FICE Concentration Detection Experiment

**FIGURE 4:**
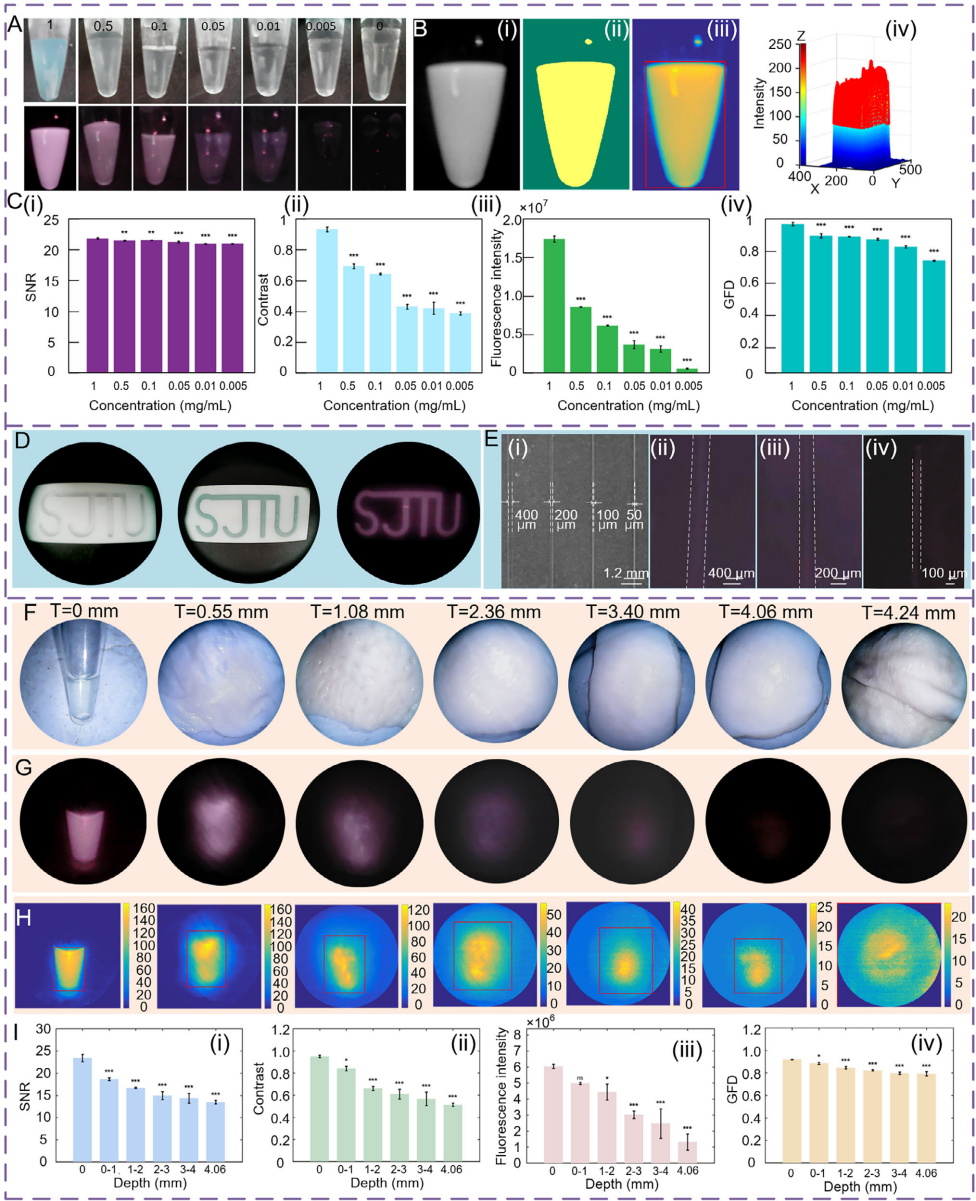
FI experiment using NIR‐II FICE. (A) WLI and NIR‐II FI conducted at concentrations of 1, 0.5, 0.1, 0.05, 0.01, and 0.005 mg mL^−1^. (B‐i) Grayscale image, (ii) binary mask image, (iii) automatically identified target region accompanied with minimum bounding rectangle, and (iv) 3D distribution map of fluorescence intensity of 1 mg mL^−1^ FNP solution. (C‐i) SNR, (ii) contrast, (iii) fluorescence Intensity, and (iv) GFD measured at different concentrations. (D) FI experiment conducted in irregular regions. (E) Minimum‐resolution imaging experiment. (F) WLI (T: thickness of porcine gastric tissue), (G) NIR‐II FI, and (H) target regions automatically identified by algorithms, along with their minimum bounding rectangles, under porcine gastric tissues of varying thicknesses. (I‐i) SNR, (ii) contrast, (iii) fluorescence intensity, and (iv) GFD of fluorescence images obtained under porcine gastric tissues of varying thicknesses.

Imaging tests were initially conducted on fluorescent nanoprobe solutions with different concentrations to study the sensitivity of NIR‐II FICE imaging. Pre‐prepared FNP solutions at the concentrations of 1, 0.5, 0.1, 0.05, 0.01, and 0.005 mg mL^−1^ were placed in a dark environment. Subsequently, the WLI and NIR‐II FI modes of NIR‐II FICE were successively activated to capture images, yielding the white‐light and fluorescence images shown in Figure 4A. The fluorescence signals were processed using MATLAB, and the data were visualized to acquire a grayscale image at a concentration of 1 mg mL^−1^ (Figure 4B‐i), a binary mask image (Figure 4B‐ii), an image with automatic target region recognition and minimum bounding rectangle selection (Figure 4B‐iii), and a 3D distribution map of fluorescence intensity (Figure 4B‐iv). The visualization processing results of fluorescence images at other concentrations can be found in Figure S7. These results indicated that the minimum detectable concentration resolution reached 0.01 mg mL^−1^, which demonstrated high detection sensitivity. Experiments were performed in triplicate. The SNR (Figure 4C‐i) of fluorescence images, the contrast between fluorescence signals and the background (Figure 4C‐ii), the fluorescence intensity (Figure 4C‐iii), and the gray‐level fluorescence density (GFD) (Figure 4C‐iv) at various concentrations can be calculated using Equations (1)—(4). Statistical significance was assessed using one‐way analysis of variance (ANOVA), with results annotated by asterisks: *p < 0.05, **p < 0.01, and ***p < 0.001. Within the concentration range of 0–1.0 mg mL^−1^, the values of the SNR, contrast, fluorescence intensity, and GFD evidently decreased as the concentration decreased. However, the SNR remained stable above 20 dB across all the tested concentrations. This finding implies that the signal consistently dominated the noise at different concentration levels, which is beneficial for the FI detection of pathological tissues. When the concentration exceeded 0.1 mg mL^−1^, the contrast between the fluorescence signal and the background reached 0.6 or greater, enabling the fluorescence signal to be more clearly distinguished from the background, thus further enhancing the accuracy of FI detection. Moreover, the GFD consistently stayed above 0.7 throughout all the tested concentrations, clearly showing that the nanoparticles displayed high luminescence efficiency and stability under different concentration conditions. These results indicated that the combination of NIR‐II FICE and FNP for imaging features a high SNR, good contrast, and stable fluorescence intensity and intensity density, which all effectively improved the imaging quality, rendering it suitable for biomedical imaging.

#### 2.3.2 NIR‐II FICE Imaging Resolution Detection Experiment

In the large‐area imaging experiment, the prepared fluorescent nanoprobe solution was injected into irregular chips with square, circular, triangular, and “SJTU”‐engraved grooves. Subsequently, imaging was performed using NIR‐II FICE. Complete fluorescent images of squares, circles, and triangles (Figure S8), along with a distinct “SJTU” pattern (Figure 4D), were successfully acquired. The results demonstrated that NIR‐II FICE, enabled by NIR‐II FNP, possesses efficient fluorescence signal capture capability, and it can accurately distinguish specific patterns within a large field of view. To evaluate the minimum planar resolution, the NIR‐II FICE system was benchmarked using linear microfluidic channels of varying widths (50, 100, 200, and 400 µm) filled with a 1 mg/mL FNP solution (Figure 4E‐i). Under controlled dark conditions, the NIR‐II FI mode of the capsule endoscope was activated for data acquisition. As shown in Figure 4Eii–iv, clear images of tiny linear regions with widths of 400, 200, and 100 µm were successfully obtained. While the 50 µm wide structures could not be clearly distinguished. These results clearly indicated that the minimum planar resolution achievable by the NIR‐II FICE imaging system is 100 µm. Such high‐resolution imaging ability has remarkable potential for the detection of early‐stage minute lesions in the gastrointestinal tract.

#### 2.3.3 Penetration Depth Experiment of NIR‐II FICE

The prepared porcine gastric tissue samples were placed on microtubes containing NIR‐II FNP, and FI was carried out to investigate the penetration capability of NIR‐II FICE into tissues (NIR‐II FI @ 2.2 V, 150 mA). Figure 4F presents the WLI results when the porcine gastric tissue coverage depths were 0, 0.55, 1.08, 2.36, 3.4, 4.06 and 4.24 mm. Figure 4G shows the corresponding NIR‐II FI results for the same tissue thicknesses, and Figure 4H depicts the pseudo‐color‐processed images where the target regions are automatically identified and captured by the algorithm, along with their minimum bounding rectangles. The SNR (Figure 4I‐i) of fluorescence images, the contrast between fluorescence signals and the background (Figure 4I‐ii), the fluorescence intensity (Figure 4I‐iii), and the GFD (Figure 4I‐iv) at various thicknesses of tissue coverage can be calculated using Equations (1)—(4). Experiments were performed in triplicate for porcine gastric tissues with different thicknesses: 0 mm (uncovered control), 0–1, 1–2, 2–3, 3–4, and 4.06 mm. Imaging results of the replicate experiments are presented in Figure S9. Statistical significance for these groups was likewise assessed using one‐way ANOVA. The tissue thickness increased, the SNR, contrast, fluorescence intensity, and GFD all exhibited a decreasing trend. Within a depth of 4.06 mm, the SNR consistently remained above 13 dB, the contrast consistently exceeded 0.5, and the GFD steadily stayed above 0.7. These findings effectively demonstrated that NIR‐II FICE retains effective imaging capability even when confronted with a 4 mm tissue depth barrier. Consequently, we defined the maximum effective penetration depth as the tissue thickness achievable under the above‐mentioned boundary conditions. NIR‐II FICE exhibited stable and superior tissue‐penetration performance, which allows for highly sensitive detection of fluorescence signals in deep tissues. This substantial advantage enhances the detection ability for submucosal lesions in the gastrointestinal tract, thus offering vital technical support for the diagnosis of early‐stage, deeply seated gastrointestinal cancer.

### 2.4 Targeted Imaging Experiment of NIR‐II FICE

**FIGURE 5:**
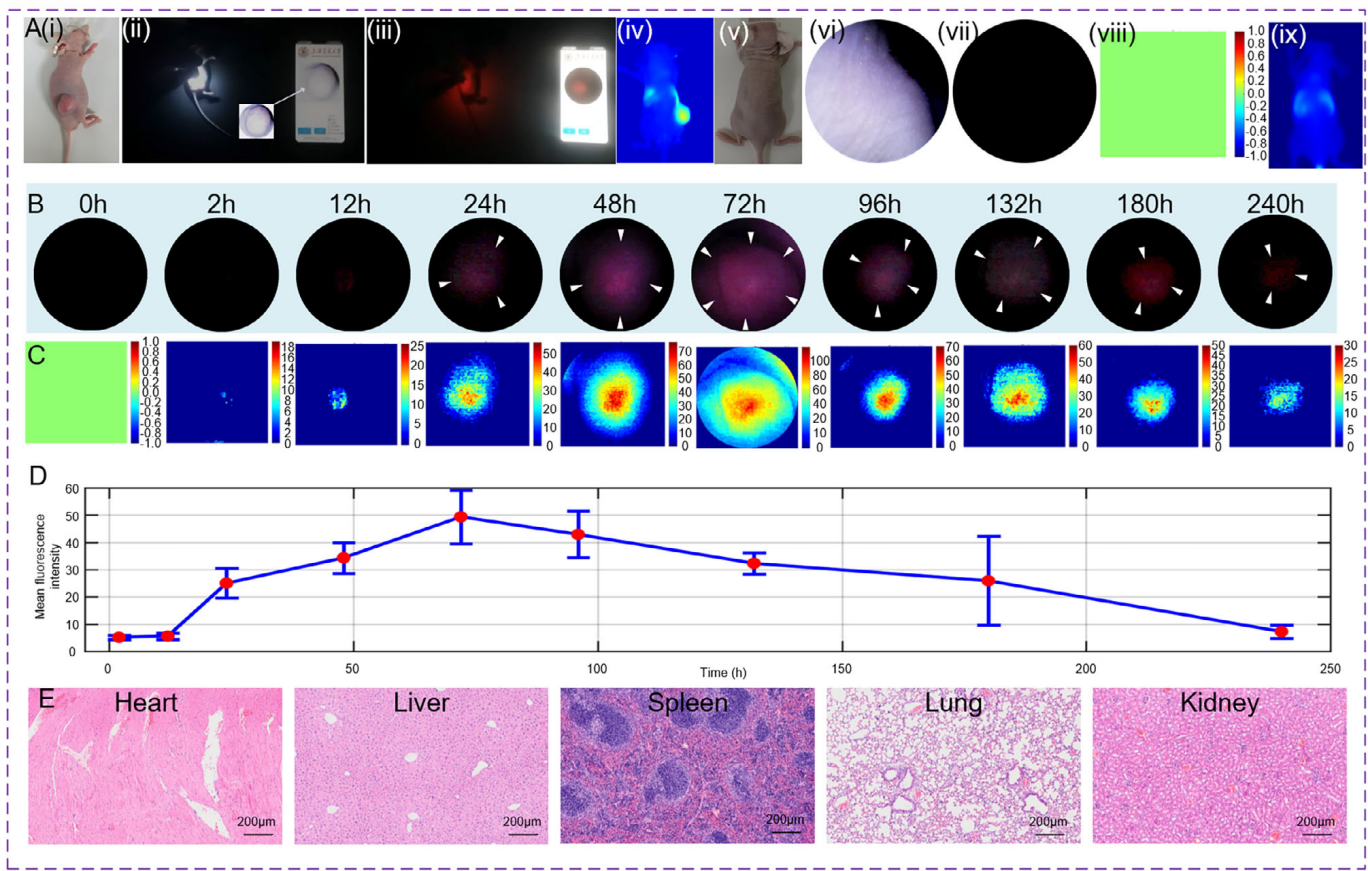
NIR‐II FICE‐targeted imaging in mice. (A)‐i) Tumor‐bearing nude mouse. (ii) WLI of mouse by using NIR‐II FICE and tumor image under white light. (iii) FI of mouse by using NIR‐II FICE. (iv) Tumor‐bearing mouse imaging under infrared imaging system. (v) Healthy control mouse. (vi) White‐light, (vii) NIR‐II fluorescence, and (vii)corresponding pseudo‐color images of a healthy mouse captured by NIR‐II FICE. (ix) Healthy control mouse imaging under in vivo imaging system. (B) FI at 0, 2, 12, 24, 48, 72, 96, 132, 180, and 240 h. (C) Pseudocolor fluorescence thermography of tumors at 0, 2, 12, 24, 48, 72, 96, 132, 180, and 240 h. (D) Time‐dependent changes in average fluorescence intensity of tumors in three mice. (E) HE staining results of the heart, liver, spleen, lungs, and kidneys in mice after the experiment.

To evaluate the targeted imaging capability of NIR‐II FICE toward cancerous tissues, in vivo imaging experiments were conducted using nude mice. FNP solutions were intravenously administered to HCT116 tumor‐bearing mice (Figure 5A‐i) and healthy control mice (Figure 5A‐v), respectively. NIR‐II FICE imaging was conducted at multiple time points (0, 2, 12, 24, 48, 72, 96, 132, 180, and 240 h). White‐light images were captured in WLI mode for both tumor‐bearing mice (Figure 5A‐ii) and healthy control mice [right hind limb, Figure 5A‐vi)]. Correspondingly, fluorescence images were acquired in FI mode to visualize the tumor‐bearing (Figure 5A‐iii,B) and healthy control groups (Figure 5A‐vii). Moreover, the location of tumor lesions in tumor‐bearing mice was accurately determined using an in vivo imaging system (Figure 5A‐iv and Figure S10). Pseudocolor processing was applied to the acquired fluorescence images to generate heatmaps, which reflect the distribution of signal intensity (Figure 5C). NIR‐II FICE, with the assistance of the FNP, evidently enabled the specific imaging of pathological tissues. Following the injection of the probe, the fluorescence signal at the lesion site gradually intensified over time, reaching its peak at specific time points (48–72 h), and demonstrated a distinct targeted enrichment effect (Figure 5B). The corresponding pseudocolor heatmaps further quantitatively confirmed this process (Figure 5C). The fluorescence signal intensity within the target regions showed a clearly defined spatial distribution, changing from weak to strong (from blue/green to yellow/red). Subsequently, as time elapsed, the signal gradually attenuated but remained detectable until 132 h. Repeated experiments were conducted on three tumor‐bearing nude mice to verify the experimental validity, as shown in Figures S11 and S12. Statistical analysis of the fluorescence intensity in the images acquired at different time points yielded the line chart presented in Figure 5D. The results demonstrated typical pharmacokinetic characteristics, where the signal increased rapidly and was subsequently followed by a gradual decline. The data acquired from the three repeated experiments exhibited minimal dispersion, which suggests outstanding reproducibility. In contrast, no significant fluorescence signals were observed in the NIR‐II or pseudo‐color images of the healthy control group (Figure 5A‐vii,viii and Figure S13]. Further observations via the in vivo imaging system revealed no significant signal accumulation in non‐hepatic regions, with the background signal maintained at a baseline level (Figure 5A‐ix). Furthermore, the probes were almost completely cleared from the body within 48 h. The results were consistent across three independent experiments (Figure S14). These results demonstrate that NIR‐II FICE enables long‐term, specific targeted imaging and monitoring of diseased tissues via NIR‐II FNPs. This quantitatively and robustly validates the efficacy and stability of the NIR‐II FICE targeted imaging technology, which holds great potential for improving the detection rate of occult lesions and enhancing the clinical accuracy of early‐stage gastrointestinal cancer screening. After the experiment was completed, HE staining was conducted on the heart, liver, spleen, lungs, and kidneys of the mice. The pathological results presented in Figure 5E demonstrated that the heart, liver, spleen, lungs, and kidneys of the mice exhibited normal tissue architecture, without any signs of toxic damage, inflammation, degeneration, or necrosis.

The potential of the NIR‐II FICE system for the early diagnosis of gastrointestinal cancer stems primarily from its superior molecular targeting, high‐sensitivity detection, and deep‐tissue imaging capabilities. Specifically, the integration of CH1055@DSPE‐PEG‐FA FNP ensures specific enrichment at lesion sites, facilitating the detection of occult micro‐lesions. From a hardware perspective, the GC0308 sensor leverages hardware‐level image signal processor (ISP) mechanisms—including dynamic gain compensation and automatic exposure—to maintain signal stability and capture faint fluorescence within the dark gastric environment. Furthermore, the 1055 nm emission light possesses strong penetrative power, enabling the identification of early‐stage, deeply infiltrating submucosal lesions that are frequently overlooked by conventional white‐light endoscopy. The synergistic fulfillment of these conditions not only validates the reliability of the system but also provides critical technical support for improving the diagnostic accuracy of early gastrointestinal cancer detection.

This study demonstrates the significant potential of NIR‐II FICE in tumor detection. The clinical translation of the CH1055@DSPE‐PEG‐FA probe is supported by a solid safety foundation: CH1055 is an organic small molecule that can be excreted via the kidneys; the carrier DSPE‐PEG is an FDA‐approved component used in various clinical drugs and exhibits excellent biocompatibility; and FA, as an endogenous small‐molecule vitamin, possesses extremely low immunogenicity. Experimental results further indicate that at an effective imaging dose of 7 mg kg^−1^, histological evaluations of the tested mice showed no abnormalities. These findings establish the theoretical and experimental groundwork for transitioning from laboratory research to clinical precision medicine. Nevertheless, clinical application still faces challenges. Moving forward, we will adhere to FDA approval protocols to conduct progressive clinical trials, ranging from micro‐dose pharmacokinetics (Phase I) to large‐scale efficacy validation (Phase II/III), while utilizing techniques such as radiolabeling to investigate its dynamic distribution and clearance rates in the human body. By optimizing the renal clearance efficiency of the probe and accumulating more GLP‐grade toxicological data, NIR‐II FICE is expected to become a powerful diagnostic tool.

### 2.5 Experimental of Synergistic Wireless Powering and Magnetic Control Systems for NIR‐II FICE in a Gastric Model

**FIGURE 6:**
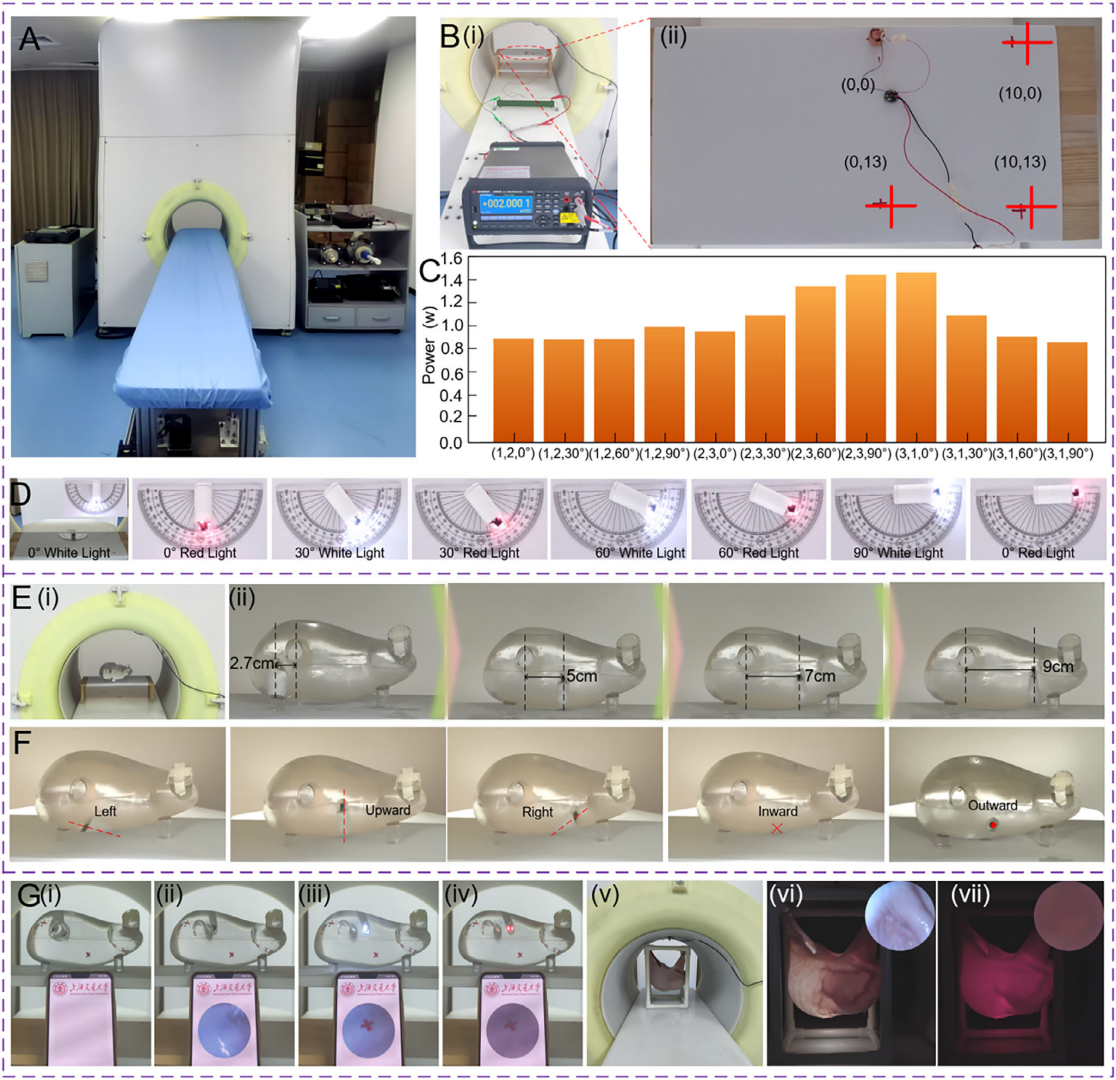
Gastric model experiment: NIR‐II FICE operating synergistically with wireless power supply and magnetic control systems. (A) Integrated wireless powering and magnetic control system with movable examination bed. (B‐i) Experimental setup for energy reception at different positions and (ii) Schematic of typical positions. (C) Energy reception data at position (0, 0) under different orientations. The labels (a, b, c) on the horizontal axis indicate the following: the a‐th dimension of the receiving coil faces the transmitting coil directly, the b‐th dimension of the receiving coil is perpendicular to the transmitting coil, and and the coil is rotated clockwise by c°. (D) WLI and FI of NIR‐II FICE at (0, 0) position when rotated counterclockwise by 0°, 30°, 60°, and 90°. (E) Translational Experiment of NIR‐II FICE on the lower wall of the gastric model. (F) Rotational experiments of NIR‐II FICE on the lower wall of the gastric model in directions: leftward, vertically upward, rightward, inward, and outward. (G‐i) NIR‐II FICE experiment on motion, positioning, image acquisition, and data transmission under synergistic wireless powering and magnetic control; (ii) connection of the mobile receiving device; (iii) motion and positioning of NIR‐II FICE; (iv) image acquisition and wireless transmission in WLI mode; (iv) image acquisition and wireless transmission in FI mode; (v) ex vivo porcine stomach experiment; (vi) image acquisition and wireless transmission in WLI mode; and (vii) image acquisition and wireless transmission in FI mode.

Wireless powering was employed to achieve the long‐term operation of NIR‐II FICE within the digestive tract. Magnetic control was used to guide NIR‐II FICE to a target position for inspection. The integrated wireless powering and magnetic control system was constructed as described in Section 4.7. It was equipped with a movable examination bed to facilitate the flexible adjustment of the patient's position during experiments. This configuration enabled effective spatial alignment between wireless energy transmission and magnetic control operations, as shown in Figure 6A.

The energy reception of NIR‐II FICE at different positions was experimentally evaluated, as shown in Figure 6B‐i. The fabricated 3D receiving coil, embedded with high‐permeability manganese‐zinc ferrite, was connected to the rectifier chip. The output power of the 3D receiving coil at angles of 0°, 30°, 60°, and 90° was experimentally measured at four positions (0, 0), (0, 13), (10, 0), and (10, 13) within the central plane of the transmitting coil (Figure 6B‐ii). The point (0, 0) represents the geometric center of the transmitting coil. Figure 6C illustrates the energy reception at this position for different orientations of the receiving coil. On the horizontal axis, each label (a, b, c) defines the orientation in the following manner: the a‐th dimension of the receiving coil is directly facing the transmitting coil, the b‐th dimension is perpendicular to it, and the whole assembly is rotated clockwise by c°. The assembled NIR‐II FICE was operated in dual mode at four different positions. Figure 6D presents WLI and FI modes at the position (0, 0) under counterclockwise rotations of 0°, 30°, 60°, and 90°, clearly demonstrating that NIR‐II FICE functioned normally in all these cases. The energy reception performance at other positions and the operational status of NIR‐II FICE in dual mode are presented in Figures S15–S17. The minimum power across different positions and angles reached 800 mW, and NIR‐II FICE functioned normally in all operational modes.

Subsequently, the motion control of NIR‐II FICE was experimentally verified. By operating the mobile control panel, the external large permanent magnet can be manipulated to move at any angle in space (Figure S18), thus controlling the motion of NIR‐II FICE that encapsulates a small permanent magnet. The designed gastric model was filled with water, and NIR‐II FICE was placed inside it (Figure 6E‐i). NIR‐II FICE was controlled by an external permanent magnet, which allowed it to translate on the lower wall (Figure 6E‐ii) and upper wall (Figure S19A) of the gastric model and to rotate (leftward, rightward, inward, and outward) on the lower and upper walls (Figure 6F and Figure S19B).

Lesions were marked in an ex vivo porcine stomach model to verify the operability of the NIR‑II FICE system under the synergistic effect of wireless power supply and magnetic actuation (Figure 6G‐i). When the integrated wireless power supply and magnetic control device was activated, a stable connection of the mobile receiving unit was established (Figure 6G‐ii). Subsequently, the NIR‑II FICE probe was magnetically navigated to the pre‑marked lesion site. Powered wirelessly, it carried out stable WLI and FI. The acquired images were then wirelessly transmitted to an external mobile receiving device (Figure 6G‐iii–iv and Figure S20). Further experiments in the ex vivo porcine stomach (Figure 6G‐v) confirmed the coordinated operation of the system (Figure 6G‐vi,(vii). Collectively, these results demonstrated that the NIR‑II FICE system exhibited excellent stability and reliability.

## 3 Conclusion

An NIR‐II FICE system with dual‐modal imaging capabilities was designed, developed, and experimentally validated for the early‐stage diagnosis of gastrointestinal cancer. Under the control of the image receiver, the CE is capable of switching between WLI and NIR‐II FI. The NIR‐II FNP was successfully constructed. With the aid of these nanoprobes, NIR‐II FICE demonstrated remarkable specificity in detection. It was capable of sensitively identifying nanoprobes at concentrations as low as 10 µg mL^−1^ and conducting imaging on large irregular regions and small 100 µm areas. In the ex vivo experiments, NIR‐II FICE achieved an imaging penetration depth of 4 mm in porcine stomach tissue, with an SNR exceeding 13 dB and a contrast higher than 0.5. The nude mouse targeting experiment further confirmed its ability to precisely localize pathological tissues. NIR‑II FICE effectively eliminated the invasiveness related to tethered endoscopy and overcame the limitations of existing CE imaging technologies. These limitations include a shallow tissue penetration depth caused by severe photon absorption and scattering, a tendency to miss deep‑seated and small lesions, and a low SNR ratio resulting from autofluorescence. It possesses the advantages of high sensitivity, high resolution, strong tissue penetration ability, a high SNR, and high‐contrast imaging. The integrated wireless powering and magnetic control system enabled a stable energy supply and motion control for NIR‐II FICE. To the best of the authors’ knowledge, this CE is the first CE worldwide to adopt NIR‐II FI technology. The NIR‐II FICE system provides a high‐quality, non‐invasive imaging solution for the early screening of gastrointestinal cancers, and it is anticipated to play a vital role in clinical diagnostics.

## 4 Experimental Materials and Design

### 4.1 Hardware Design

#### 4.1.1 Circuit Board and Packaging Design

The core component of NIR‐II FICE is the PCB. All components, such as the light source module, image acquisition module, microcontroller module, wireless signal transmission module, and power module, are integrated onto a foldable rigid‐flex PCB. The voltage‐regulated PCB is designed as a separate rigid PCB to avoid interference with imaging caused by high‐frequency switching signals. These modules work in collaboration to enable the functionality of NIR‐II FICE. Detailed specifications and functional descriptions of each module are provided in Note S1.1. The principle of minimal packaging was followed when designing the PCB of CE to ensure CE has compact dimensions, enabling patients to swallow it effortlessly and allowing it to pass through the digestive tract smoothly. In this process, the use of 0201 and 0402 packaged resistors, capacitors, and inductors was prioritized (Figure S21). The NIR‐II FICE shell mainly comprises an optical dome and the rest of the body. The optical dome, which demands high light transmittance, was fabricated from polycarbonate material, whereas the remaining portion was constituted by acrylonitrile‐butadiene‐styrene plastic. Both materials exhibit excellent chemical stability and corrosion resistance.

#### 4.1.2 Optoelectronic Module Design

The optical front‐end of NIR‐II FICE consists of an illumination module and an image acquisition module. The illumination module primarily comprises three broadband white‐light LEDs and two customized near‐infrared LEDs with a center wavelength of 780 nm, all of which are integrated onto a rigid annular PCB. The NIR‐II FICE employs a GalaxyCore GC0308 CMOS image sensor (1/6.5‐inch, pixel size: 3.2 µm × 3.2 µm), featuring a spectral response range of 400–1200 nm. To counteract the degradation of quantum efficiency in silicon‐based sensors at longer wavelengths, dynamic gain compensation, automatic exposure control, brightness calibration, and color balance control mechanisms were implemented by configuring the hardware‐integrated ISP. This configuration ensures that analog and digital gains are automatically activated in low‐fluorescence environments, interlocked with denoising parameters. Regarding the optical design, the NIR‐II FICE is equipped with a wide‐angle lens (FOV: 100°, aperture: F2.4) and a custom high‐specification band‐stop filter (OD > 4, transmittance > 90%) to achieve precise separation of 400–650 nm white light and 900–1200 nm fluorescence signals. To completely eliminate interference from the 780 nm excitation light, a “fourfold light‐shielding system”—comprising physical adhesive sealing, structural shielding, spectral filtering, and gap sealing—was constructed to ensure the acquisition of high‐quality NIR‐II raw data.

### 4.2 Software Design

An Android application was developed to facilitate the operation of CE by medical professionals and patients and enable real‐time image viewing. This application is capable of displaying and controlling the imaging functions of CE in real time, which enables users to adjust the shooting mode. Any Android device with this application installed is capable of serving as an image receiver, and the specific operation interface is presented in Figure S22.

### 4.3 Preparation of NIR‐II FNP

CH1055 was loaded into nanoparticles composed of DSPE‐PEG‐FA by using ultrasonic nanoprecipitation. In brief, DSPE‐PEG‐FA (5.0 mg) and CH1055 (1.0 mg) were dissolved in 10 mL of deionized water and 1 mL of DMF, respectively. Then, the DMF solution was added dropwise to the deionized water solution under vigorous ultrasound, followed by sonicating for 10 min. The unloaded CH1055 and DMF were removed by centrifuging the supernatant solution at 15 000 rpm. Finally, an appropriate amount of ultrapure water was added and sonicated to form CH1055@DSPE‐PEG‐FA and then stored at 4°C. The content of CH1055 was determined by absorption spectroscopy.

### 4.4 Characterization and Measurement

The emission spectra of the 780 nm LED, white LED, and NIR‐II FNP, as well as the absolute quantum yield of the FNP, were characterized using a fluorescence spectrometer (FLS1000, Edinburgh Instruments, UK) equipped with a calibrated integrating sphere. The absorption spectra of NIR‐II FNP were determined using an NIR spectrophotometer (Lamda 950). DLS spectrum and zeta potential were recorded on a Brookhaven Omni particle size analyzer. The morphology and structure of the NIR fluorescent probes were characterized by means of a scanning electron microscope (Thermo Scientific, US, Talos F200X G2).

### 4.5 Data Analysis

The following formulas were employed to evaluate the SNR, contrast, fluorescence intensity, and GFD of the acquired images:

(1) $$\mathrm{SNR}=20\cdot \mathrm{lo}g_{10}\left(\frac{\mu }{\sigma }\right)$$

where *µ* represents the mean pixel value of the fluorescent signal region, and *σ* represents the standard deviation of the pixel values in the background noise region;

(2) $$\mathrm{Contrast}=\frac{\bar{\mathrm{ROI}}-\bar{\mathrm{Background}}}{\bar{\mathrm{ROI}}+\bar{\mathrm{Background}}}\;$$

where $\bar{\mathrm{ROI}}$ and $\bar{\mathrm{Background}}$ represent the mean pixel values of the region of interest and the background region, respectively;

(3) $$\mathrm{Fluorescence}\;\mathrm{intensity}\;=\mathrm{Sum}\left(\mathrm{ROI}\right)$$

where Sum(ROI) denotes the total sum of pixel values within the NIR‐II fluorescence region extracted using the binary mask; and

(4) $$\mathrm{GFD}\;=\frac{\mathrm{Sum}\left(\mathrm{ROI}\right)}{\mathrm{Sum}\left(\mathrm{Minimum}\;\mathrm{external}\;\mathrm{rectangle}\right)}\;$$

where Sum(Minimum external rectangle) represents the sum of the total pixel values within the minimum rectangular region.

### 4.6 Cytotoxicity Evaluation

#### 4.6.1 CCK‐8 Activity Assay

CCK‐8 activity assay was carried out on GES‐1 to assess the biocompatibility of NIR‐II FNP. GES‐1 cells were seeded in 96‐well plates at a density of 4 × 10^3^ cells per well and cultured overnight. Then, the medium was replaced by fresh medium containing varying concentrations of CH1055@DSPE‐PEG‐FA, and the cells were incubated for an additional 24 h. Afterwards, the cells were washed three times using PBS and supplemented with fresh medium, and the standard CCK‐8 assay was performed to determine the relative cell viability.

#### 4.6.2 Live/Dead Staining Fluorescence Observation

Cell viability assay was conducted to evaluate the impact of the white‐light and NIR light sources of NIR‐II FICE on cell viability. Human gastric mucosa GES‐1 cells were cultured. Subsequently, those cells in the logarithmic growth phase and that exhibited a good growth status were selected and seeded at a density of 1 × 10^5^ cells per well in a confocal cell culture dish. The culture was set to last for 24 h. The cells were subsequently divided into three groups. The first group functioned as the control group, which did not undergo any light irradiation. By contrast, the second and third groups were exposed to white‐light LED and NIR light sources for 30 min, respectively After the irradiation process was completed, the live/dead working solution was added to the cells, which were then incubated for 30 min. The cells were rinsed with PBS, followed by an incubation period at room temperature for 30–45 min. The staining working solution was aspirated to halt the incubation. Finally, the labeled cells were observed under a confocal microscope.

### 4.7 Integrated Wireless Power Supply and Attitude Control Device

An integrated wireless power supply and magnetic control device was developed to ensure the long‐term operation of NIR‐II FICE within the digestive tract and precisely control its position and orientation. The electromagnetic induction‐based wireless power supply system comprises two components: the transmitting end and the receiving end. The transmitting end comprises a DC power supply, a capacitor, an inverter PCB, and a transmitting coil (Figure S23); the receiving end consists of a 3D receiving coil and a rectifier and voltage regulator PCB (Figure S24). Detailed information can be found in Supporting Information (Note S1.2). The magnetic control system consists of a neodymium‐iron‐boron (NdFeB) permanent magnet, which has a diameter of 10 mm and a height of 5 mm and is installed inside the CE, and an externally‐mounted NdFeB permanent magnet with a diameter of 120 mm and a height of 180 mm (Figure S25). Both permanent magnets are nickel‐plated and axially magnetized to ensure that their magnetic fields exhibit rotational symmetry along the axis. A mechanical structure was constructed to drive the movement of the external permanent magnet (Figure S26). Additionally, an electronic control cabinet with a movable control panel was installed to operate the motor (Figure S27), which enables precise control of NIR‐II FICE. Detailed information can be found in Supporting Information (Note S1.3). Moreover, a movable bed was installed to cooperate with the wireless power supply and magnetic control system, enabling NIR‐II FICE to receive energy stably and move within the human digestive tract (Figure S28).

### 4.8 Animal Model

HCT116 cells cultured under standard conditions and in the logarithmic growth phase were digested, centrifuged, and resuspended. Subsequently, the cell concentration was adjusted to 5 × 10^6^ cells mL^−1^. A 100 µL cell suspension was subcutaneously injected into the right hind limb of three 8‐week‐old nude mice (approximately 25 g each). Another three healthy nude mice without any treatment served as the control group. All animals were obtained from Shanghai Lingchang Biotechnology Co., Ltd. (China). All animal experiments were approved by the Animal Research Management Committee of Shanghai Jiao Tong University (Approval No. A2025236). The tumor size and body weight were monitored every 2 days. At no point did the tumor volume exceed 2000 mm^3^ or 10% of the body weight. Detailed monitoring data are provided in Figures S29 and S30 and Tables S1 and S2.

### 4.9 NIR‐II FICE Imaging Experiment

#### 4.9.1 NIR‐II FICE Nanoprobe Concentration Detection Experiment

The prepared NIR‐II fluorescent nanoprobe solution was diluted to the following concentrations: 1, 0.5, 0.1, 0.05, 0.01, and 0.005 mg mL^−1^. Subsequently, it was imaged using NIR‐II FICE.

#### 4.9.2 NIR‐II FICE Resolution Test

Square, circular, triangular, and irregular chips featuring SJTU pattern were designed and fabricated. Subsequently, NIR‐II FICE was utilized to perform large‐area imaging of the NIR‐II fluorescent nanoprobe solution in irregular regions. Microfluidic chips equipped with line pillars having diameters of 50, 100, 200, and 400 µm were designed and fabricated for the minimum resolution detection imaging experiment.

#### 4.9.3 NIR‐II FICE Penetration Depth Detection Experiment

NIR‐II FI depth detection experiments were conducted ex vivo on porcine gastric tissue. A pre‐prepared solution of FNP was injected into a 10 µL microtube and then embedded with Landin gel, enabling the porcine gastric tissue to be spread flat onto the tube. Fifteen porcine stomach tissue samples, with thicknesses ranging from 0 mm to 5 mm, were prepared. Specifically, three samples were included in each of the following thickness ranges: 0–1 (with thicknesses of 0.4, 0.5, and 0.61 mm), 1–2 (1.08, 1.24, and 1.32 mm), 2–3 (2.12, 2.36, and 2.42 mm), 3–4 (3.05, 3.2, and 3.4 mm), and 4–5 mm (4.06, 4.24, and 4.8 mm). Subsequently, these samples were sequentially placed over the microtube and imaged using NIR‐II FICE.

#### 4.9.4 In Vivo Targeted Imaging of Nude Mice by Using NIR‐II FICE

A 200 µL solution of the FNP was intravenously injected into tumor‐bearing nude mice and the healthy control mice, with an equivalent probe concentration of 7 mg kg^−1^ in the mice. This was established based on a series of preliminary experiments and a comprehensive review of the existing literature [40, 41]. Subsequently, NIR‐II FICE was utilized to record the fluorescence images of the mice at 0, 2, 12, 24, 48, 72, 96, 132, 180, and 240 h after injection. The tumor location was determined using an imaging system (IVIS Spectrum, PerkinElmer) as a control. The parameters were set as follows: excitation at 808 nm, emission > 900 nm, and an exposure time of 50 ms.

## Author Contributions

**Weicheng Wang**: conceptualization, methodology, investigation, validation, data curation, writing – original draft, and project administration. **Jinlei Jiang**: validation, data curation, and supervision. **Zhengting Wang**: investigation and validation. **Shuqi** Liu: validation. **Wei Wang**: visualization. **Xi Chen**: validation. **Qirui Zhao**: software. **Xinyuan Cui**: validation. **Cheng Zhou**: investigation, validation, visualization, and writing – review & editing. **Shengsheng Cui**: investigation, validation, visualization, annd writing – review & editing. **Daxiang Cui**: conceptualization, investigation, resources, project administration, funding acquisition, and writing – review & editing.

## Conflicts of Interest

The authors declare no conflicts of interest.

## Supporting information




**Supporting File**: advs75532‐sup‐0001‐SuppMat.docx.

## Data Availability

The data that support the findings of this study are available on request from the corresponding author. The data are not publicly available due to privacy or ethical restrictions.
