# Supplementary material for: Wireless, Deep‐Seeing Smart Pill: A NIR‐II Fluorescence Imaging Capsule Endoscope for Gastrointestinal Cancer
Source: Adv Sci (Weinh). 2026 May 12;13(42):e75532. doi: 10.1002/advs.75532 (PMC13335670; doi:10.1002/advs.75532)
Supplement: Supplementary file 1 — Supporting File: advs75532‐sup‐0001‐SuppMat.docx. [file ADVS-13-e75532-s001.docx]

Supporting Information for

**Wireless, deep-seeing smart pill: A NIR-II fluorescence imaging capsule endoscope for gastrointestinal cancer**

Weicheng Wang et al.

Corresponding author: zhou-cheng@sjtu.edu.cn; [cuisheng@sjtu.edu.cn](mailto:cuisheng@sjtu.edu.cn); dxcui@sjtu.edu.cn

The PDF file includes:

Legends for the hardware circuit board design of the NIR-II FICE.

Legends for the wireless power supply module.

Legends for the attitude control module.​​

Figs. S1 to S30.

S1 Supplemental Notes

S1.1 Hardware circuit board design of the NIR-II FICE.

The image acquisition module consists of a commercial complementary metal-oxide-semiconductor (CMOS) image sensor (3.2 mm × 2.7 mm) capable of Video Graphics Array (VGA) resolution (640 × 480), a lens, a lens mount, and a band-stop optical filter with a diameter of 4.7 mm and a thickness of 2 mm. The CMOS image sensor utilized is a camera-chip device developed by GalaxyCore Microelectronics Co., Ltd., with a resolution of 640 × 480 pixels. The sensor is configured using a microcontroller and the I²C protocol and transmits data via the digital video port (DVP) interface connected to the microcontroller’s digital camera interface. The CMOS image sensor is soldered onto a circular printed circuit board (PCB), and its geometric center is precisely aligned to the center of the circular PCB. The lens mount is firmly fixed to the PCB around the CMOS image sensor with an opaque black ultraviolet adhesive, guaranteeing coaxial alignment with the CMOS image sensor. The lens is mounted on the lens base through a threaded knob.

The microcontroller functions as the core processing unit of the capsule endoscopy system and handles functional coordination and task scheduling. The system enables efficient data exchange between modules via an SPI bus architecture, ensuring the systemic execution of core functions, such as image acquisition, data processing, and wireless transmission. The master control chip utilizes the STM32H743AII6 microprocessor (7 mm × 7 mm package), which integrates an ARM Cortex M7 core and a hardware JPEG encoding module to support the real-time compression of YCbCr4:2:2 raw images. In the design of the communication protocol, the system utilizes a virtual I^2^C bus implemented through GPIO pins to configure the parameters of the CMOS sensor. The visual data link directly links the DVP port to the DCMI vision channel of the processor, transmitting image signals via an 8 bit data bus (D0–D7).

This hardware architecture utilizes the DMA transfer mechanism to facilitate the high-speed loading of image data. Consequently, it effectively reduces the utilization of CPU resources and offers reliable data support for subsequent image processing and wireless transmission modules. The capsule endoscopy system utilizes Bluetooth Low Energy (BLE) to establish the wireless transmission unit, facilitating reliable communication with the master control unit through an SPI bus. The integrated chip is packaged in a 3.5 mm × 3.6 mm WLCSP, integrates a dual-core architecture (comprising a BLE protocol stack and a Cortex M4 processor), operates at the 2.4 GHz ISM frequency band, and supports a 2 Mbps BLE data transmission rate. Its transmission power ranges from −20 to +8 dBm. In the maximum transmission power mode, it can achieve stable communication through human tissues, and the effective transmission distance satisfies the data return requirements between the capsule and external receiver.

In the power module design, this prototype utilizes two switching power supply chips (MPM3804) in QFN-10 packaging (2 mm × 2 mm). Through resistor trimming, one MPM3804 module outputs 2.8 V to provide power for the CMOS image sensor and white-light LED, whereas the other MPM3804 module outputs 2.2 V to supply power to the microcontroller, wireless communication chip, and LEDs with a central wavelength of 780 nm. A contactless switch circuit activated by an infrared light source was built using two infrared photodiodes, one N-channel enhancement–mode metal-oxide-semiconductor field-effect transistor (MOSFET), one P-channel enhancement-mode MOSFET, and several resistors. The circuit is activated when the photodiode is exposed to external infrared radiation for over five seconds, continuously connecting the power supply module to the power input port of the capsule endoscopy prototype and subsequently powering on the prototype to start its operation. According to the programmed logic, a high-level signal is automatically provided via the GPIO port. This mechanism allows the contactless switch circuit to remain conductive even after the external infrared illumination stops. The prototype is powered off by simply controlling the GPIO port through the image receiver to send a low-level signal to the contactless switch circuit. This signal blocks the conduction between the power supply module and the power input port of the capsule endoscopy prototype, thus causing the prototype to enter the shutdown state.

S1.2 Wireless power supply module

Given that the NIR-II FICE consumes a considerable amount of power when integrating light source, image acquisition, controller, and wireless signal transmission modules, we adopted a wireless power supply system to ensure the capsule endoscope’s prolonged gastrointestinal tract examination capability. Two schemes that consider the stability of the transmission power can be adopted: one uses a three-dimensional (3D) transmit coil and a one-dimensional (1D) receive coil and the other uses a 1D transmit coil and a 3D receive coil. The 3D orthogonal receive coil is characterized by simple control and stable transmission power. Among Helmholtz coils, solenoids, solenoid pairs, and multi-segment solenoids, the 1D Helmholtz transmit coil exhibits the optimal magnetic field uniformity. Therefore, the wireless power supply module of this prototype employs the configuration with a 1D transmit coil and a 3D orthogonal receive coil.

The wireless energy transmitter is composed of a DC voltage source (UDP8305M and UDP8303M), an inverter circuit PCB, fixed and adjustable capacitors, and a transmit coil. The UDP8305M generates two square-wave signals with opposing phases and has a 50% duty cycle, a frequency of 100 kHz, and a voltage amplitude of 15 V. The full-bridge inverter circuit is composed of four N-channel enhancement–mode MOSFETs (IRFR4615). Specifically, two MOSFETs are combined to form a half-bridge circuit, which converts the DC voltage generated by the inverter circuit into AC voltage. Fixed and adjustable capacitors are connected in series with the coil. When the capacitive reactance is equal to the inductive reactance of the coil, the coil current attains its maximum value and delivers optimal output energy to the capsule endoscope. The output increases with the voltage of the DC voltage source (UDP8303M). In this study, the output voltage is 27 V, and the output current is 4 A. The transmitting coil adopts a Helmholtz coil structure, which is wound with 250 strands of enameled copper wire in 80 turns. Taking into account the power consumption and dimensional requirements during examinations, we configured the coil frame to have a diameter of 52 cm. The width of each individual coil is 10 cm.

The wireless energy receiver is composed of a series resonant circuit and a rectifier-regulator circuit. The rectifier-regulator circuit comprises three rectifiers (BAS4002) and one voltage regulator chip (LT8609). The series resonant circuit is composed of a 3D receive coil, which is composed of three mutually orthogonal 1D coils and three sets of tuning capacitors that facilitate resonance. Each coil is wound with a single strand of copper wire. The 3D receive coil integrates high-permeability manganese–zinc ferrite as a magnetic core. This integration enhances the mutual inductance between the transmit and receive coils, consequently leading to a substantial increase in the induced power of the receive coil. The fabricated 3D receive coil prototype is presented in Figure S24. First, the framework for coil winding is designed using SolidWorks. Once fabricated, the customized manganese–zinc ferrite core material is inserted into the framework. Subsequently, three sets of coils with mutually perpendicular axes are wound on the framework. The first dimension (innermost layer) is composed of 14 layers with a total of 321 turns, which are wound with 0.12 mm-diameter copper wire. The second dimension (middle layer) is composed of 14 layers, with 301 turns. It is wound with the same type of copper wire. The third dimension (outermost layer) consists of six layers with 355 turns and wound with the same type of copper wire.

S1.3 Attitude control module

The active control technology of capsule endoscopes has attracted considerable research. Compared with internally driven capsule endoscopes, which consume a high amount of energy and have complex mechanical configurations susceptible to mechanical damage, externally driven systems leverage the force exerted by an external magnetic field on a small internal magnet within the capsule endoscope to control its motion. This method not only eliminates the need for an additional power supply but also allows for the miniaturization of the capsule endoscope. The magnetic field has a minimal impact on human health, guaranteeing safe operation. Moreover, the magnetic permeability of the human body is approximately equal to that of a vacuum, preventing the distortion of the magnetic field distribution. As a result, this prototype serves as a magnetic control device that manipulates the movement of the capsule endoscope within the stomach.

The motion control module consists of two permanent magnets: an external permanent magnet (EPM) for generating the external magnetic field and an internal permanent magnet (IPM) integrated inside the capsule. Given the size limitations of the capsule endoscopy prototype, the IPM is a cylindrical neodymium–iron–boron (NdFeB) magnet with a diameter of 10 mm, height of 5 mm, and performance grade of N52. The EPM is a cylindrical NdFeB magnet with a diameter of 120 mm and a height of 180 mm, with a performance grade of N35. Both magnets have their nickel-plated surfaces and are axially magnetized, generating a spatial magnetic field with rotational invariance around their axes. The movable bed is controlled by the Y-axis servo motor. The motion control module consists of an electrical control cabinet. According to electrical wiring standards, a power module, a drive module, and a main control module are installed inside a metal cabinet. The power module is classified into 220V AC and 24V DC, which supply power to different modules. The drive module consists of five servo drivers, which are used to independently drive one of the five servo motors along the X, Y, Z, U, and V axes. The core of the main control module is a programmable logic controller (PLC). The touch screen is connected to the electrical control cabinet via a wired connection and communicates solely with the PLC. Users can input parameters to the PLC through the touch screen. Subsequently, the PLC issues positioning signals to the servo drivers via a program, which activates the servo motors to drive the EPM to adjust to the designated position and posture. After the servo motors complete the positioning action, they send feedback signals to the PLC, which in turn transmits the real-time position and posture information of the EPM to the touch screen for display and enables users to monitor the status.


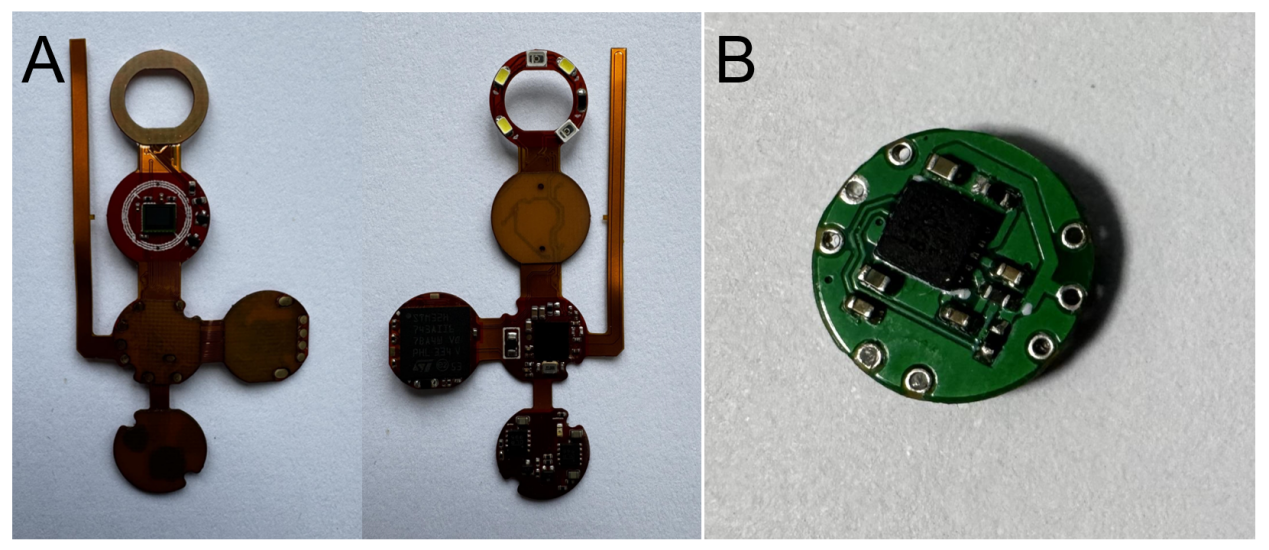


Figure S1. (A) Front and back sides of the rigid-flex board. (B) Voltage-stabilizing circuit board.


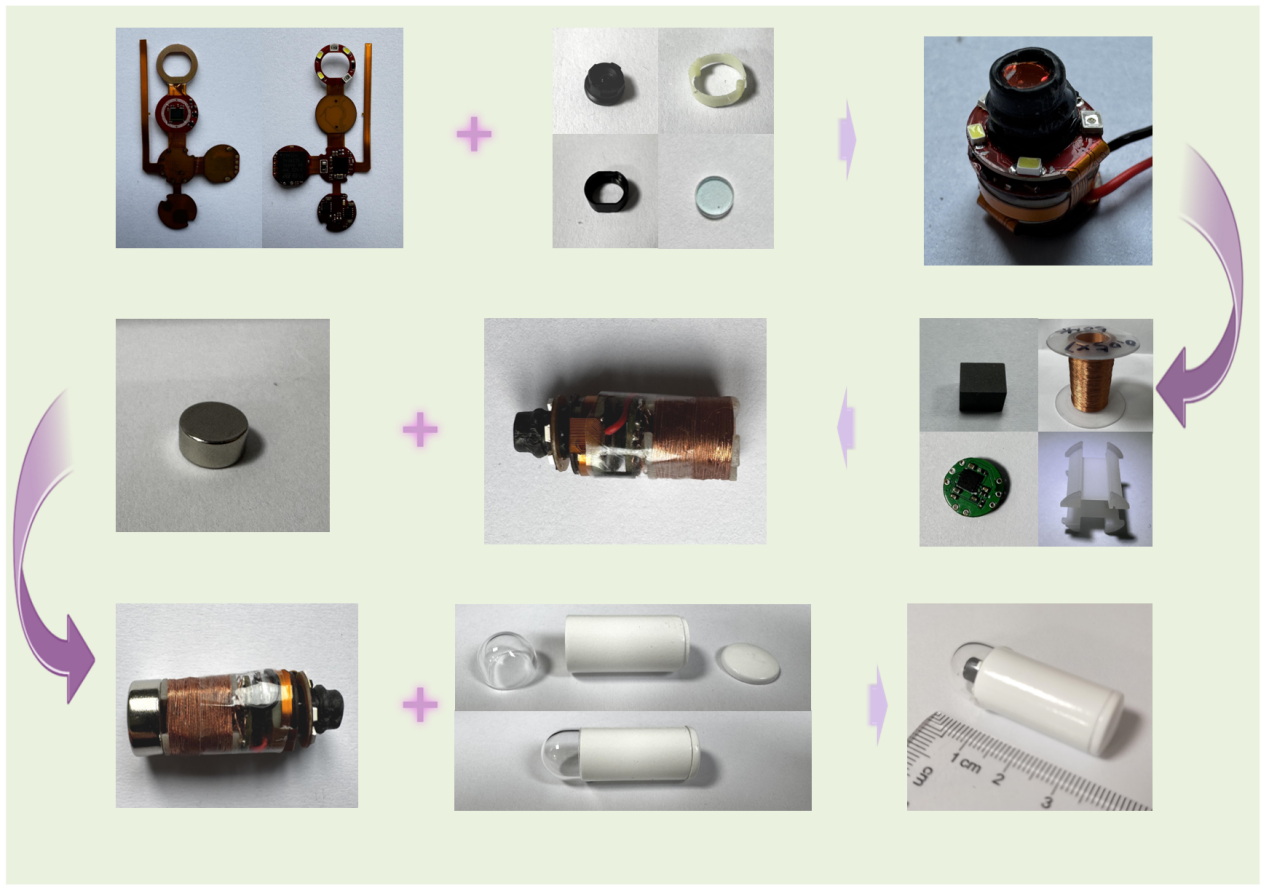


Figure S2. Flowchart of capsule endoscope assembly.


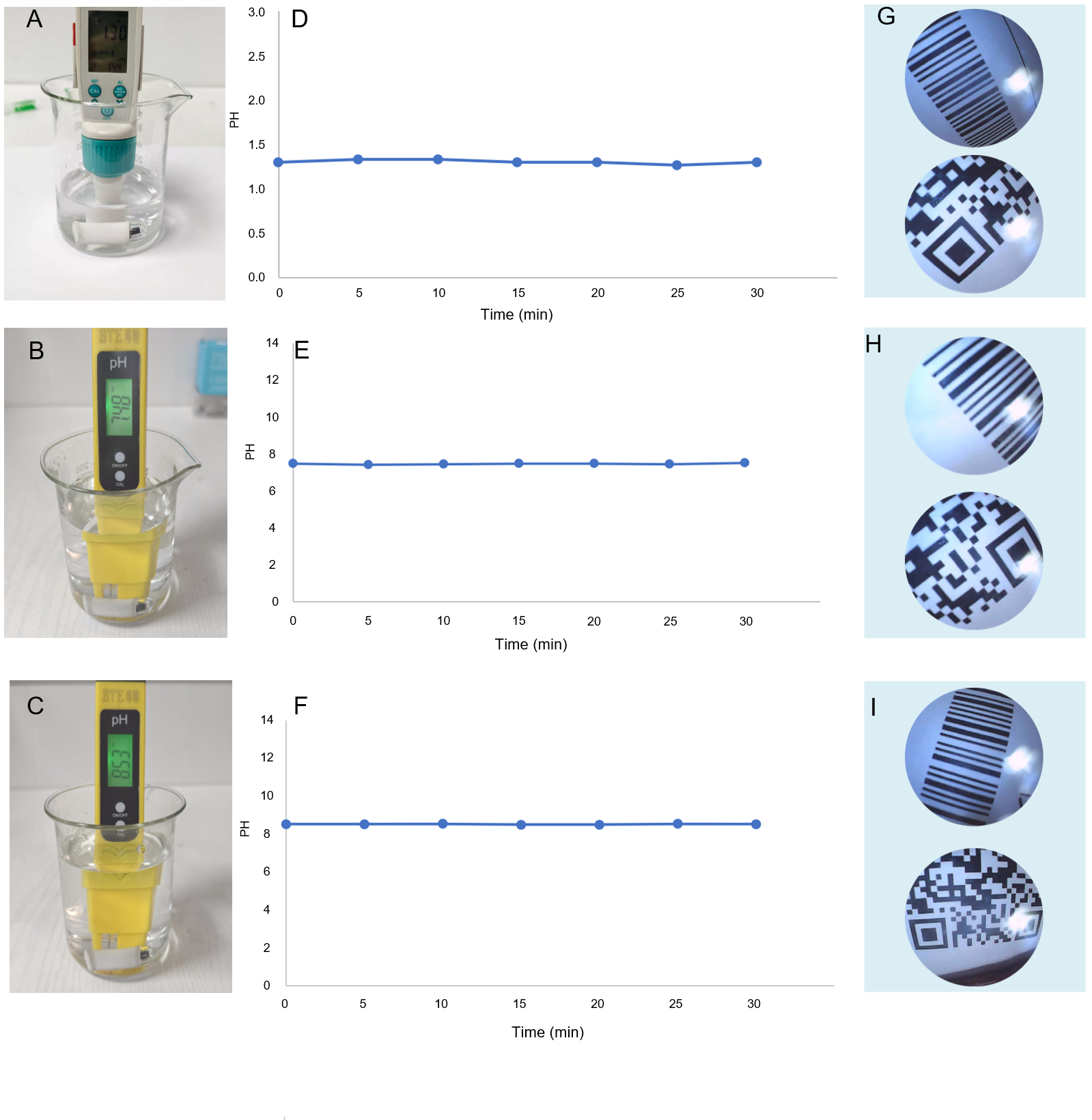


Figure S3. (A) FICE was tested in artificial gastric fluid. (B) FICE was tested in pure water. (C) FICE was tested in baking soda water. (D) The change in the pH value of artificial gastric fluid within 30 minutes. (E) The change in the pH value of pure water within 30 minutes. (F) The change in the pH value of baking soda water within 30 minutes. (G) The imaging results obtained after FICE was immersed in artificial gastric fluid for 30 minutes. (H) The imaging results obtained after FICE was immersed in pure water for 30 minutes. (I) The imaging results obtained after FICE was immersed in baking soda water for 30 minutes.


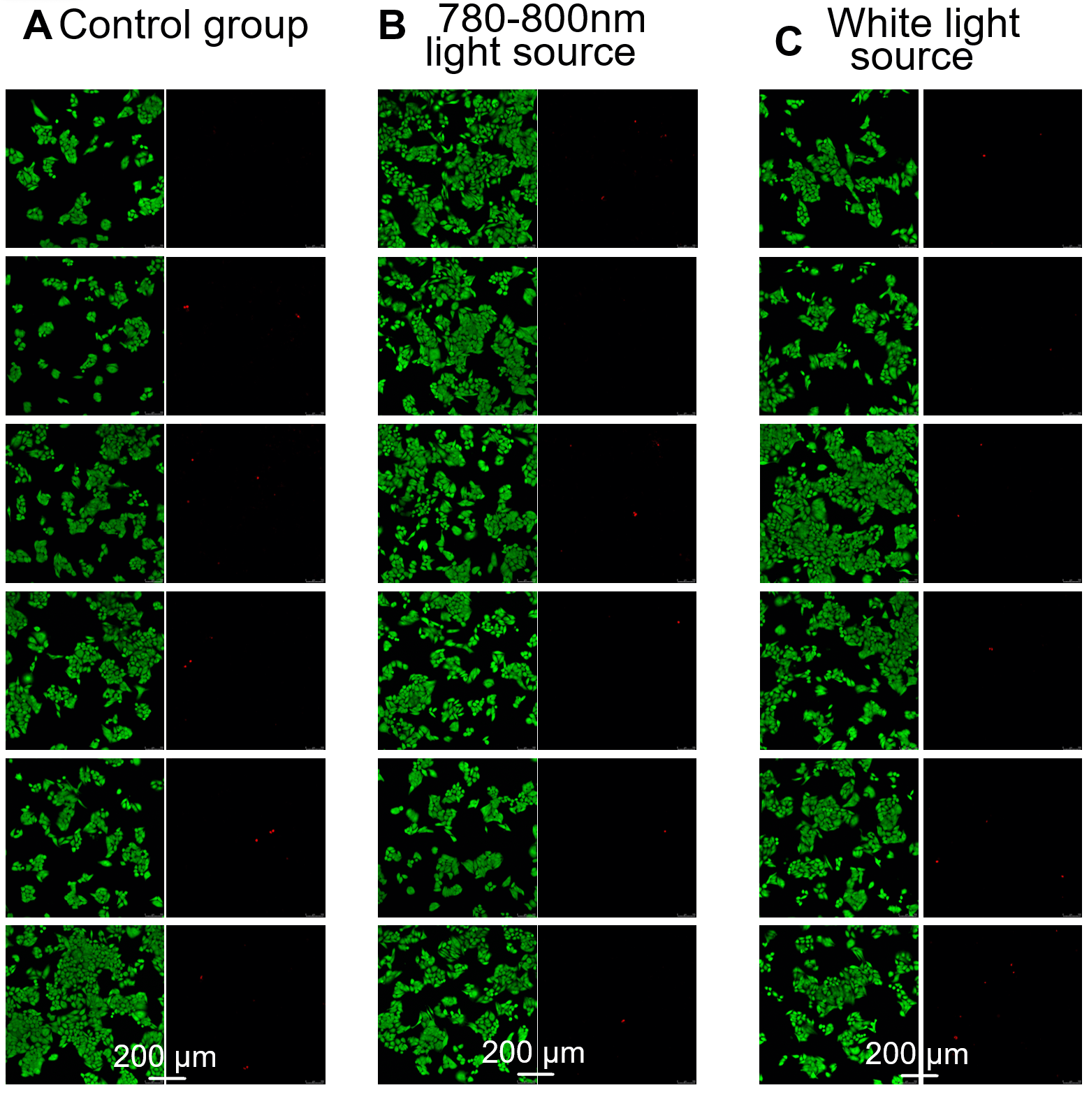


Figure S4. (A) Confocal images of live-dead staining in the control group. (B) Confocal live and dead cell images under near-infrared light source excitation. (C) Confocal live and dead cell images under the excitation of a white light source


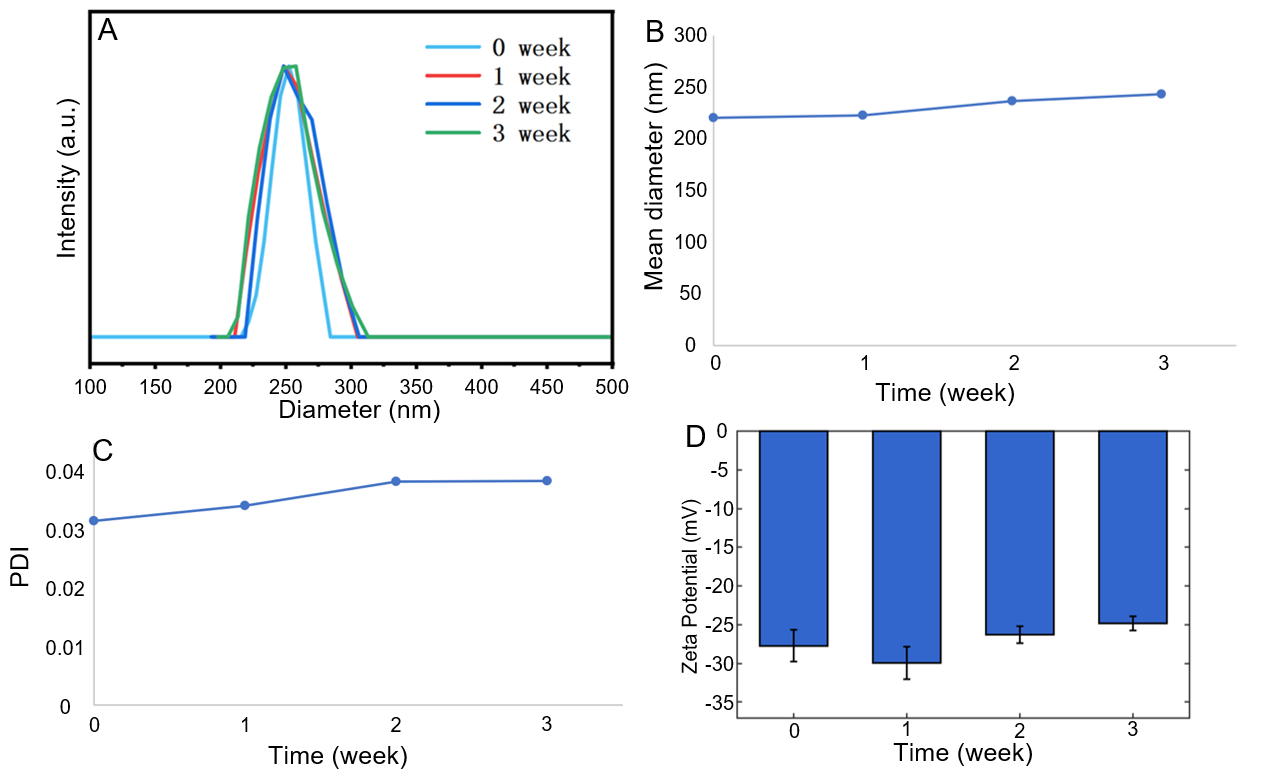


Figure S5. (A). NIR-II fluorescent nanoprobe particle size distribution. (B). The average particle size change of the NIR-II fluorescent nanoprobe within three weeks. (C). The change in PDI of NIR-II fluorescent nanoprobe within three weeks. (D). The changes in the Zeta potential of the NIR-II fluorescent nanoprobe within three weeks.


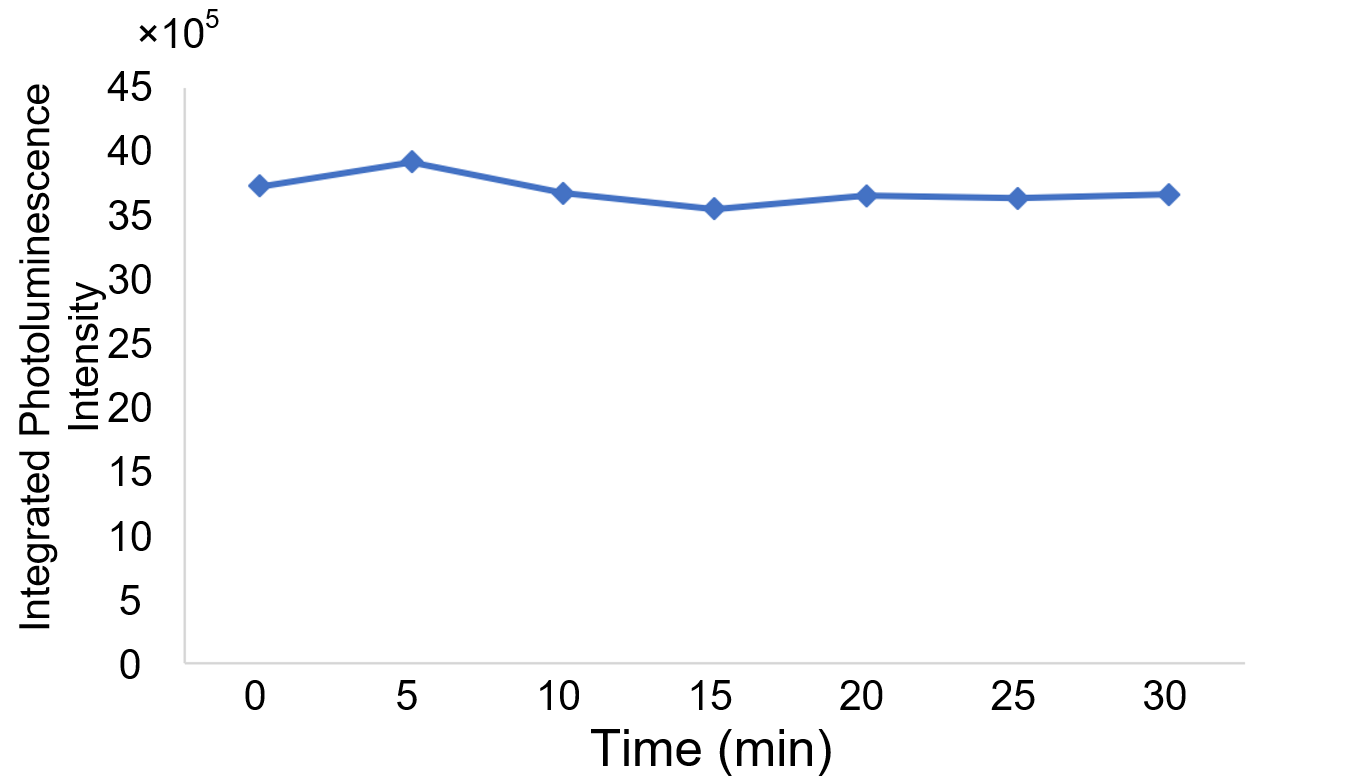


Figure S6. Change curve of integrated fluorescence intensity derived from different emission spectra.


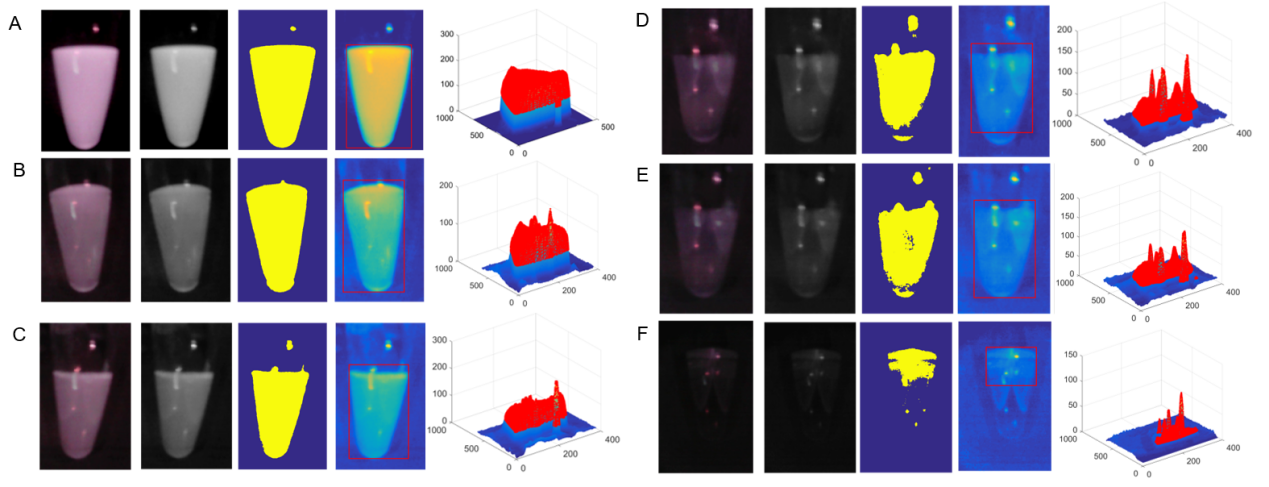


Figure S7. Original images, grayscale images, binary mask images, automatically identified target regions, minimum bounding rectangle annotations, and 3D fluorescence intensity distribution maps of nanoprobe solution samples at concentrations of (A) 1, (B) 0.5, (C) 0.1, (D) 0.05, (E) 0.01, and (F) 0.005 mg/ml.


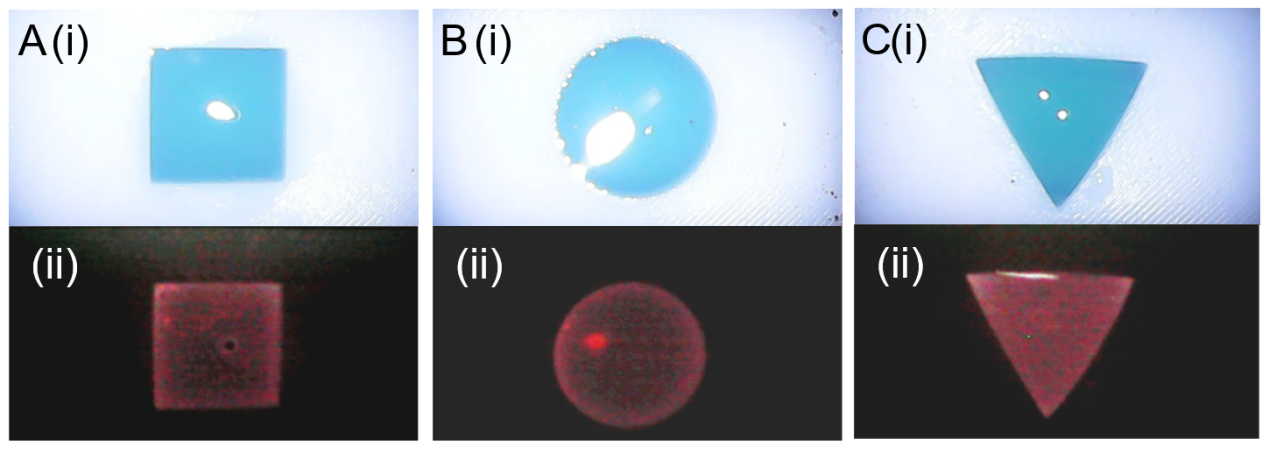


Figure S8. (A) Nanoprobe-injected square chip: (i) white-light image and (ii) fluorescence image. (B) Nanoprobe-injected circular chip: (i) white-light image and (ii) fluorescence image. (C) Nanoprobe-injected triangular chip: (i) white-light image and (ii) fluorescence image.


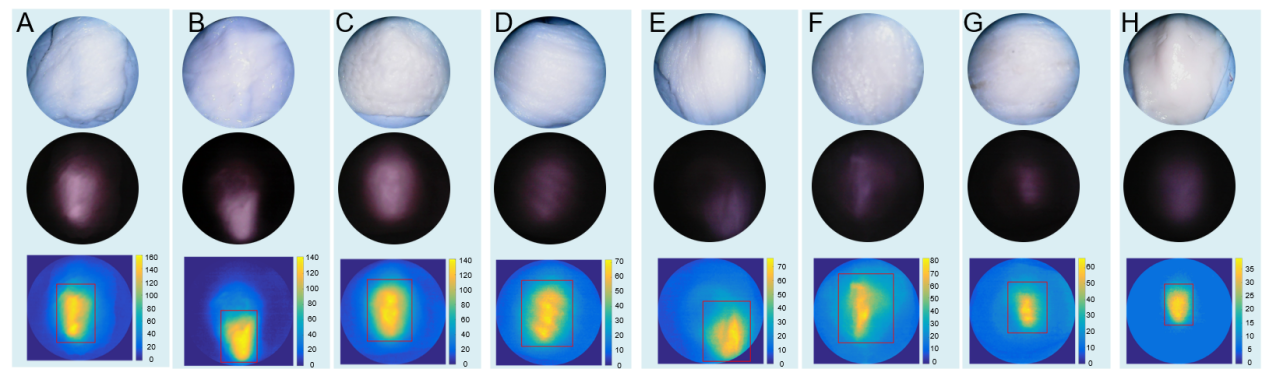


Figure S9. White-light imaging, fluorescence imaging, automatically identified target regions, and minimum bounding rectangle annotations of samples with thicknesses of (A) 0.4 mm, (B) 0.61 mm, (C) 1.24 mm, (D) 1.32 mm, (E) 2.12 mm, (F) 2.42 mm, (G) 3.05 mm, and (H) 3.2 mm.


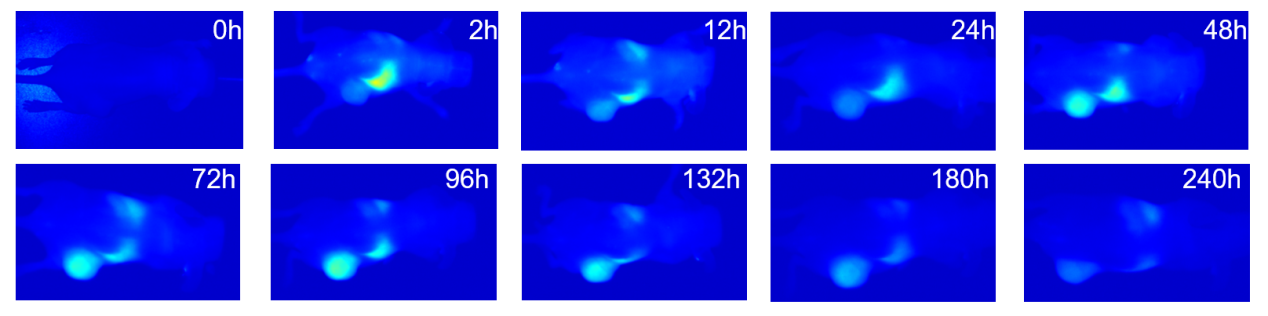


Figure S10. ​Infrared imaging of mice at different time points.


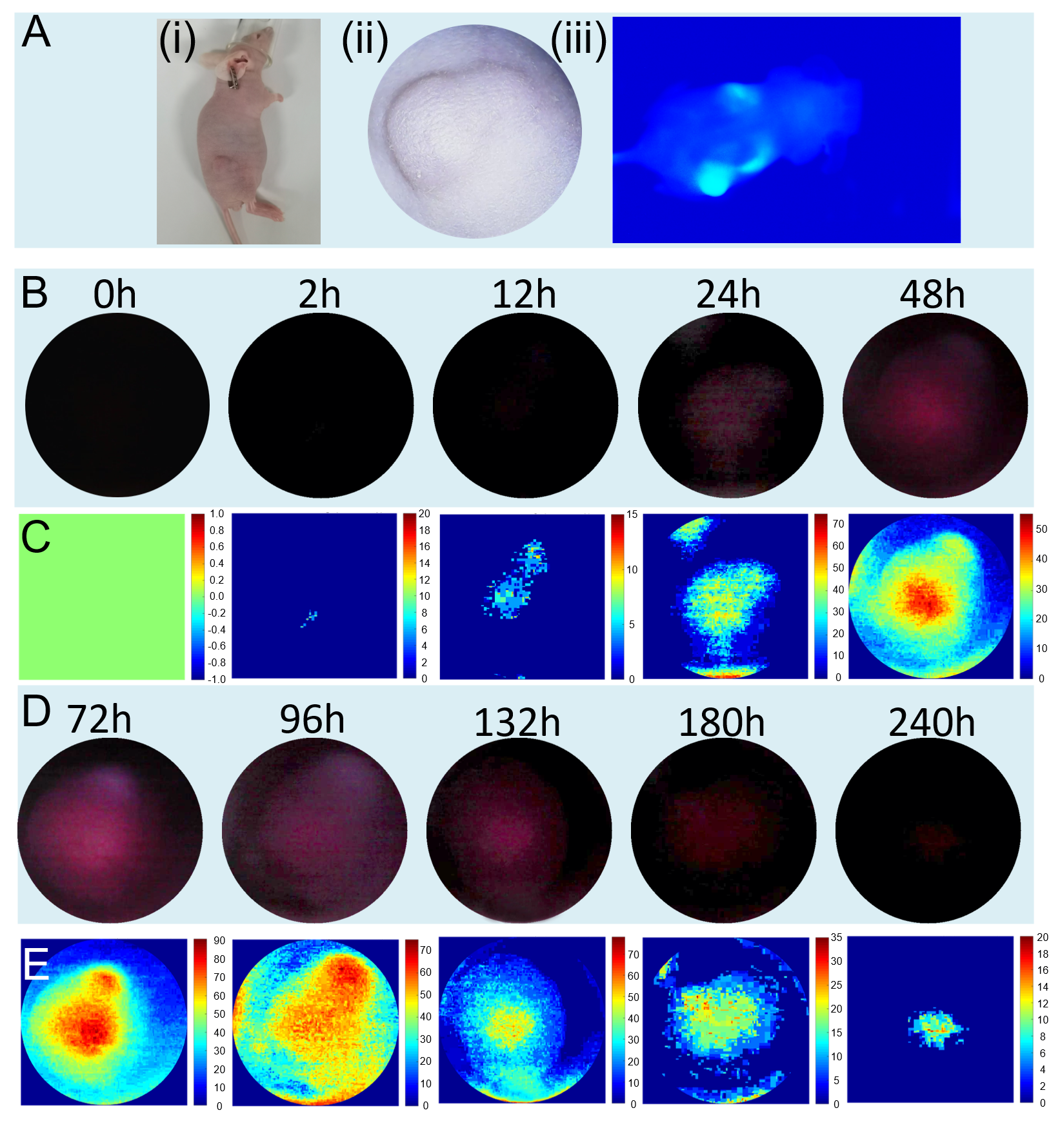


Figure S11. NIR-II FICE imaging experiment of mice 2. (A) (i) Nude mouse. (ii) White light imaging of tumors with NIR-II FICE. (iii) Mouse imaging under the infrared imaging system. (B) Fluorescence imaging at 0, 2, 12, 24, and 48 h of tumors. (C) Pseudocolor display of tumors fluorescence imaging at 0, 2, 12, 24, and 48 h. (D) Fluorescence imaging at 72, 96, 132, 180, and 240 h of tumor. (E) Pseudocolor display of tumor fluorescence imaging at 72, 96, 132, 180, and 240 h.


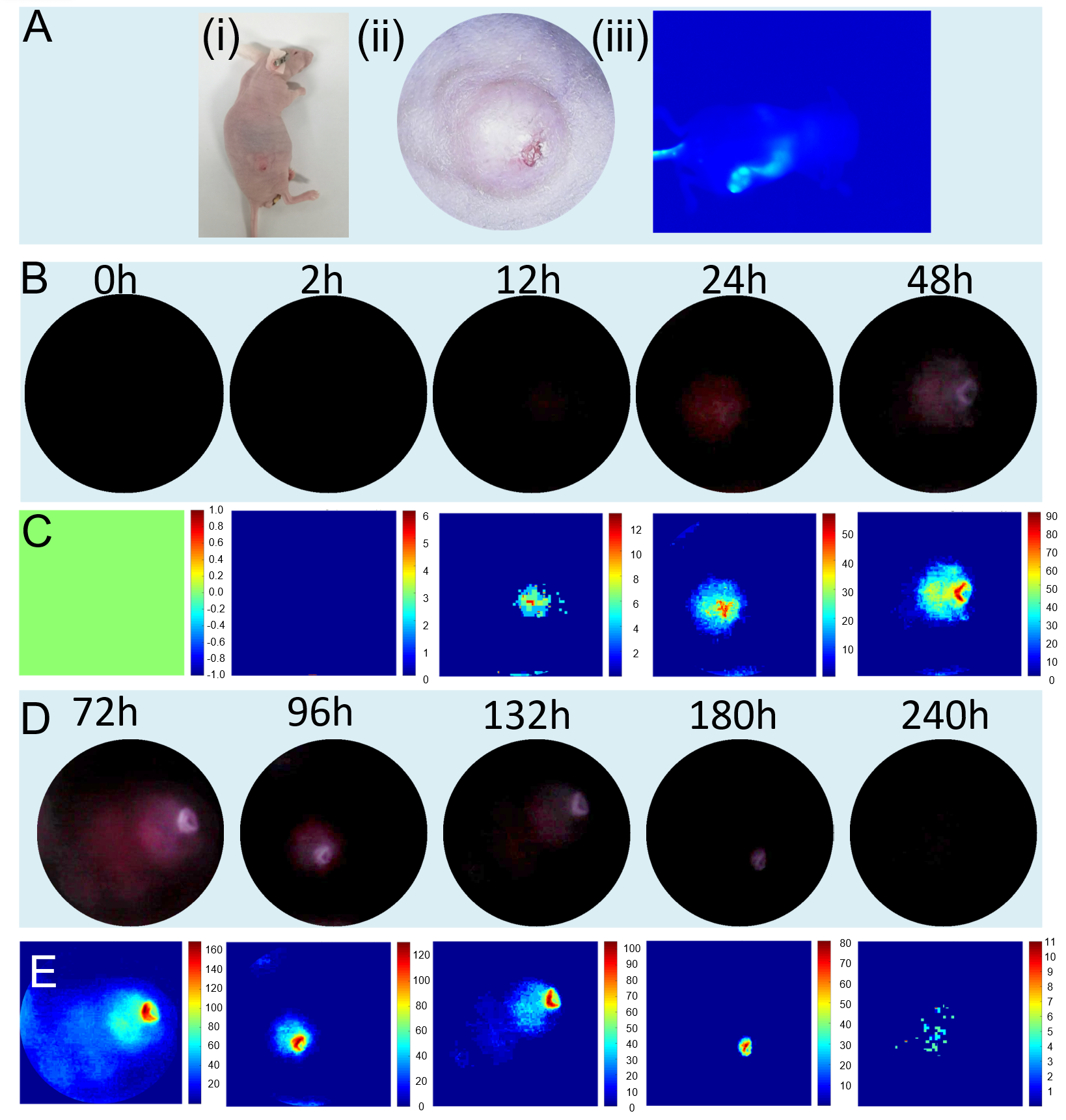


Figure S12. NIR-II FICE imaging experiment of mice 3. (A) (i) Nude mouse. (ii) White-light imaging of tumors with NIR-II FICE. (iii) Mouse imaging under the infrared imaging system. (B) Fluorescence imaging of tumors at 0, 2, 12, 24, and 48 h. (C) Pseudocolor display of tumors fluorescence imaging at 0, 2, 12, 24, and 48 h. (D) Fluorescence imaging of tumor at 72, 96, 132, 180, and 240 h. (E) Pseudocolor display of tumor fluorescence imaging at 72, 96, 132, 180, and 240 h.


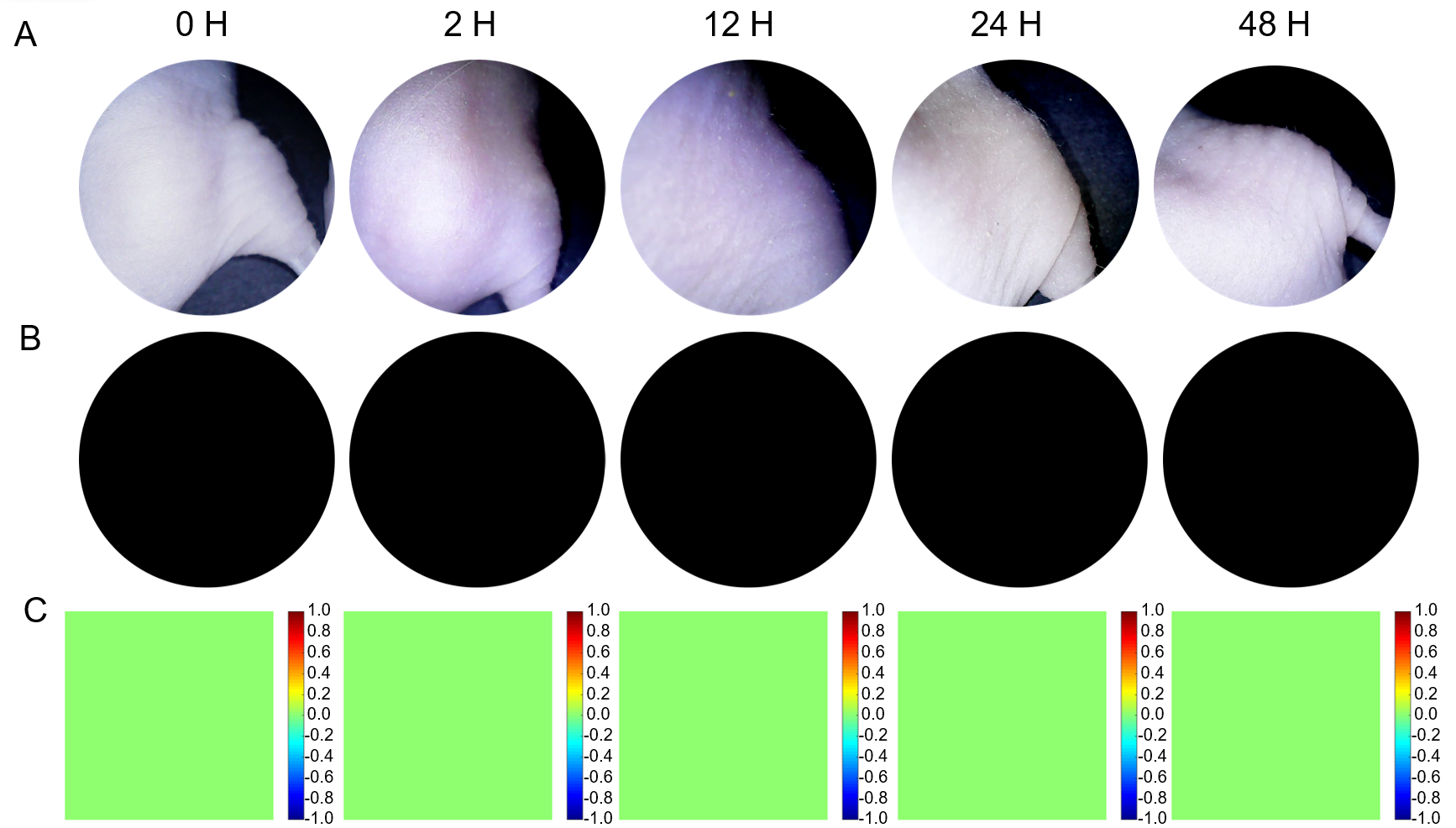


Figure S13. (A) White light images, (B) NIR-II fluorescence images, and (C) corresponding pseudo-color images of the right hind limb of mice at 0, 2, 12, 24, and 48 h post-injection, captured using the NIR-II FICE.


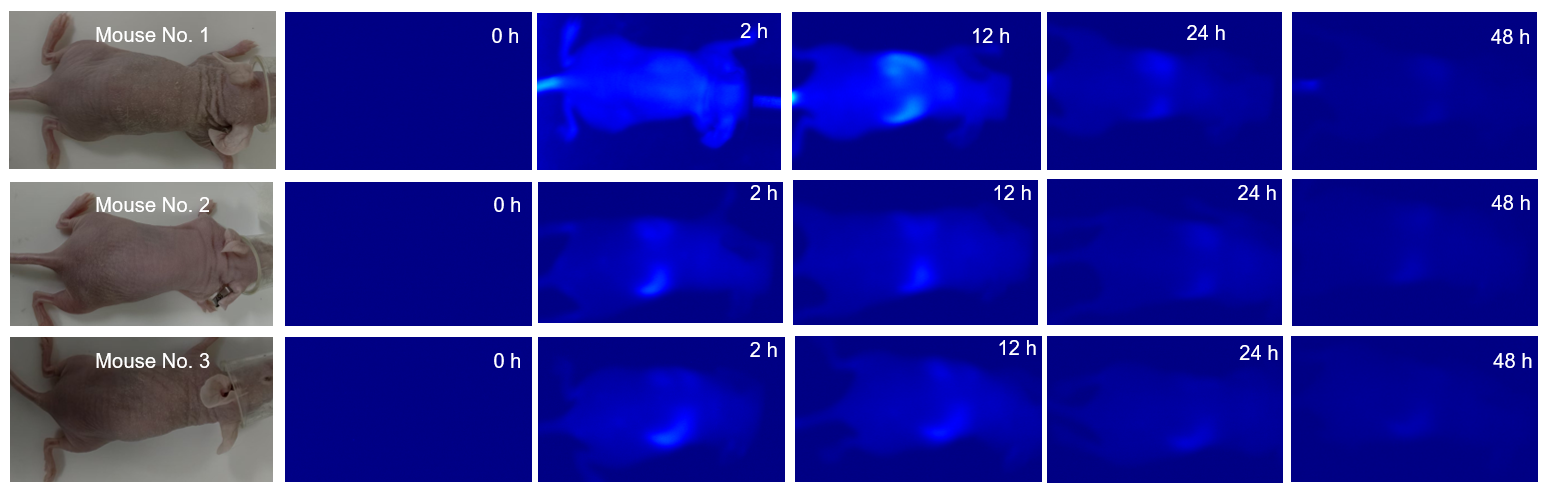


Figure S14. NIR-II fluorescence images were recorded at 0, 2, 12, 24, and 48 h using the IVIS Spectrum system (PerkinElmer; excitation power: 5 W, exposure time: 50 ms).


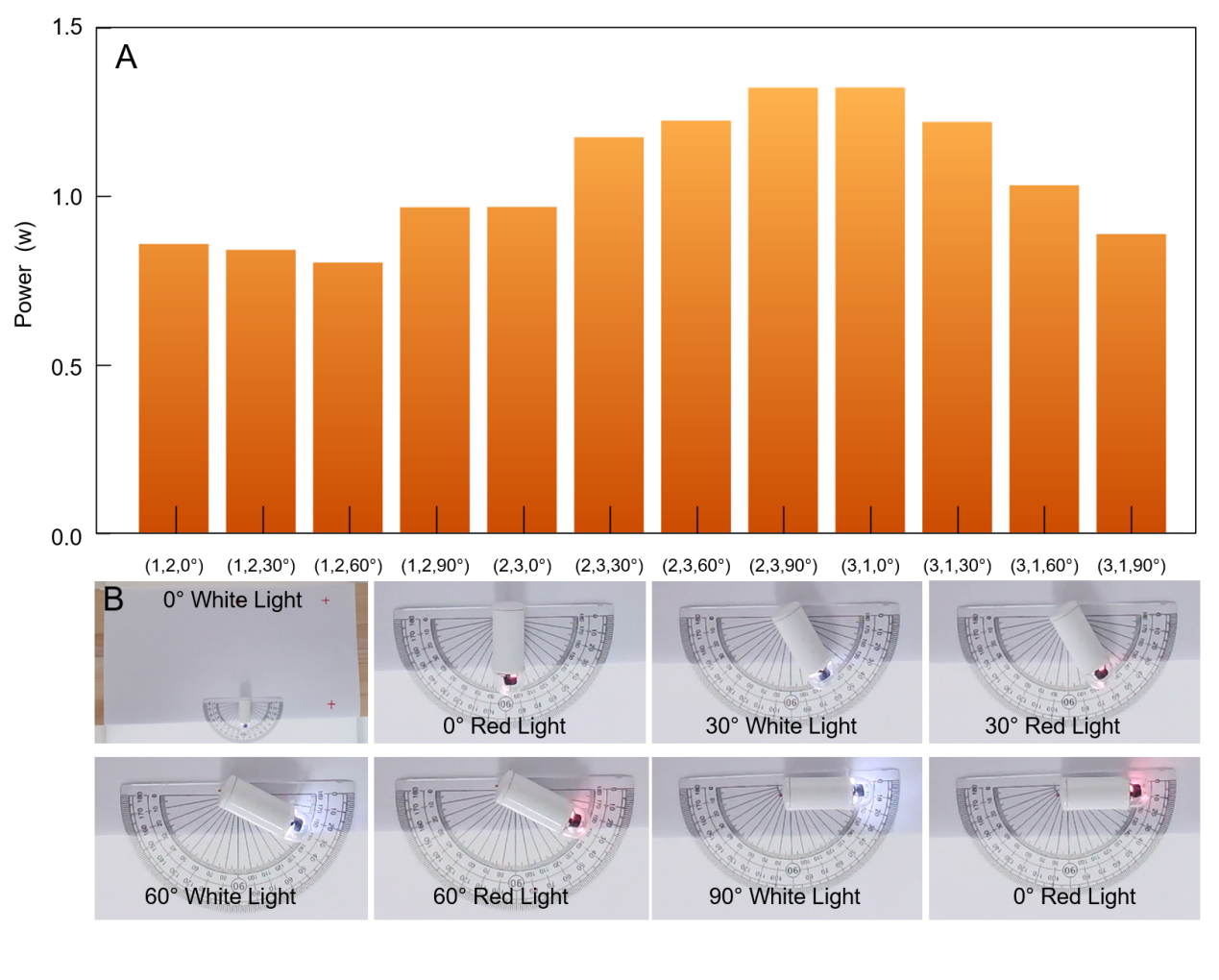


Figure S15. (A) Energy reception data at position (0, 13) under different postures. The horizontal coordinates (a, b, c) indicate that the a-th dimension of the receive coil is aligned to the transmit coil, the b-th dimension of the receive coil is laterally oriented relative to the transmit coil, and the receive coil is rotated clockwise by c°. (B) White-light and fluorescence imaging of the position (0, 13) under counterclockwise rotations of 0°, 30°, 60°, and 90°.


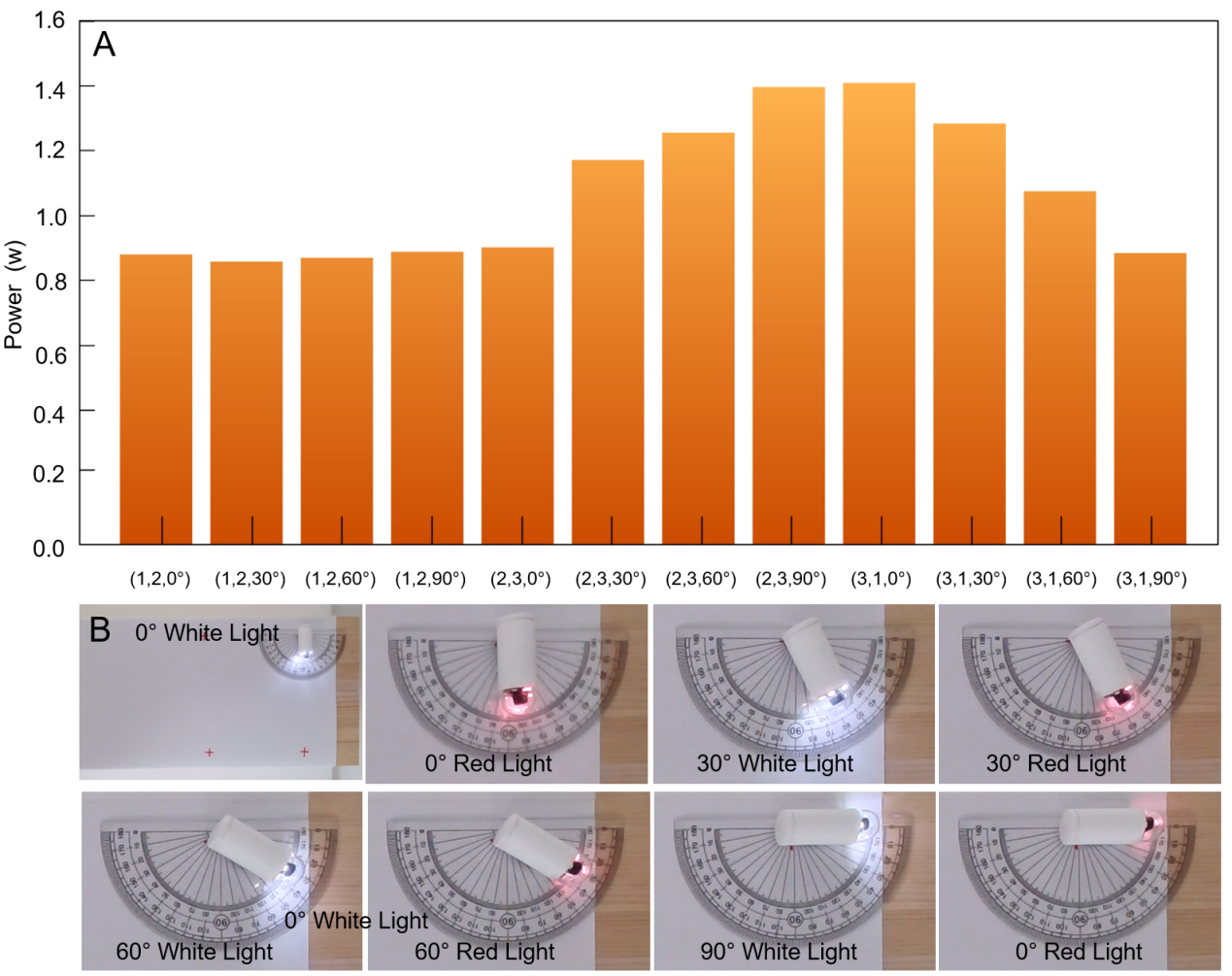


Figure S16. (A) Energy reception data at position (10, 0) under different postures. The horizontal coordinates (a, b, c) indicate that the a-th dimension of the receive coil is aligned with the transmit coil, the b-th dimension of the receive coil is laterally oriented relative to the transmit coil, and the receive coil is rotated clockwise by c°. (B) White-light and fluorescence imaging of the position (10, 0) under counterclockwise rotations of 0°, 30°, 60°, and 90°.


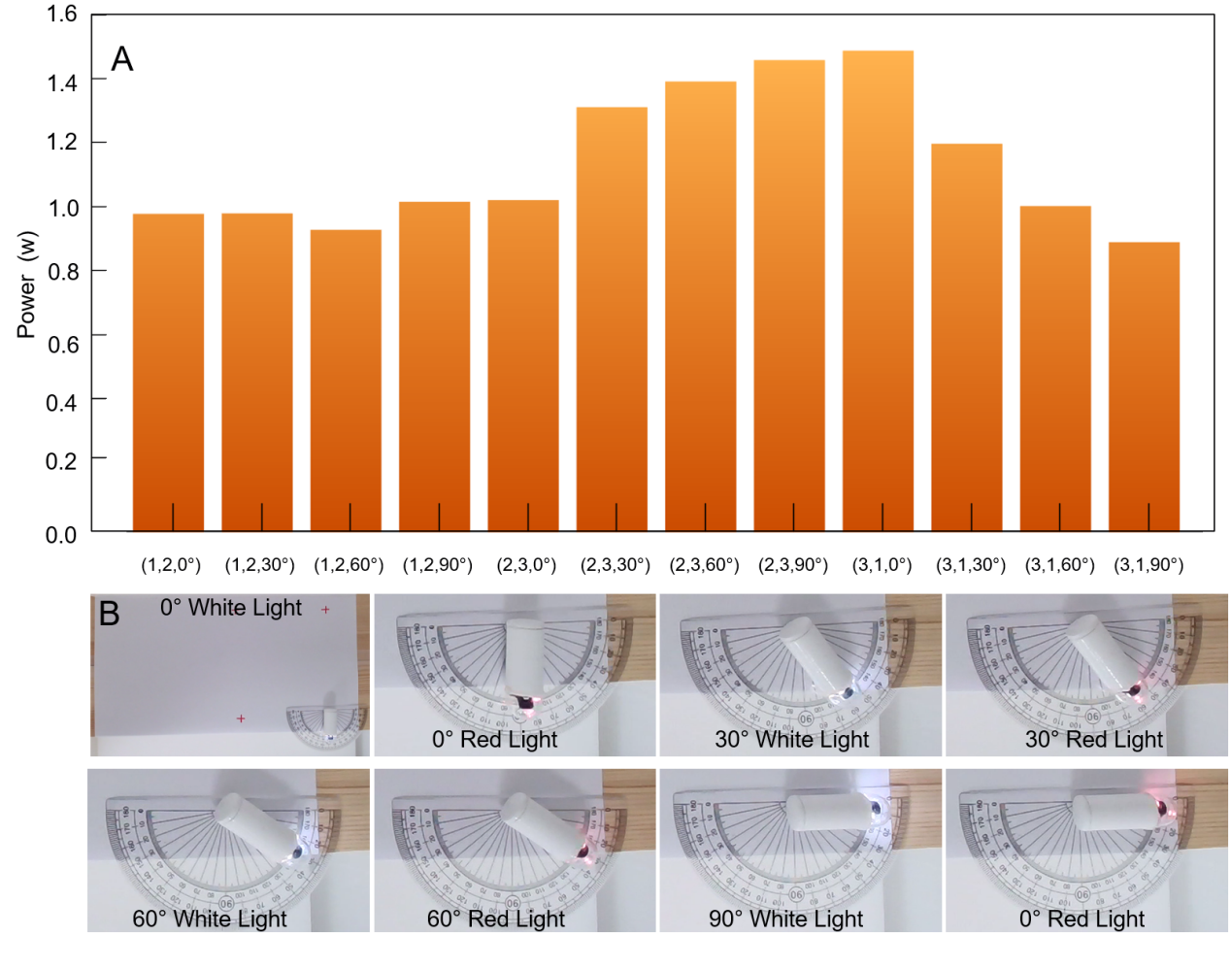


Figure S17. (A) Energy reception data at position (10, 13) under different postures. The horizontal coordinates (a, b, c) indicate that the a-th dimension of the receive coil is aligned to the transmit coil, the b-th dimension of the receive coil is laterally oriented relative to the transmit coil, and the receive coil is rotated clockwise by c°. (B) White-light and fluorescence imaging of the position (10, 13) under counterclockwise rotations of 0°, 30°, 60°, and 90°.


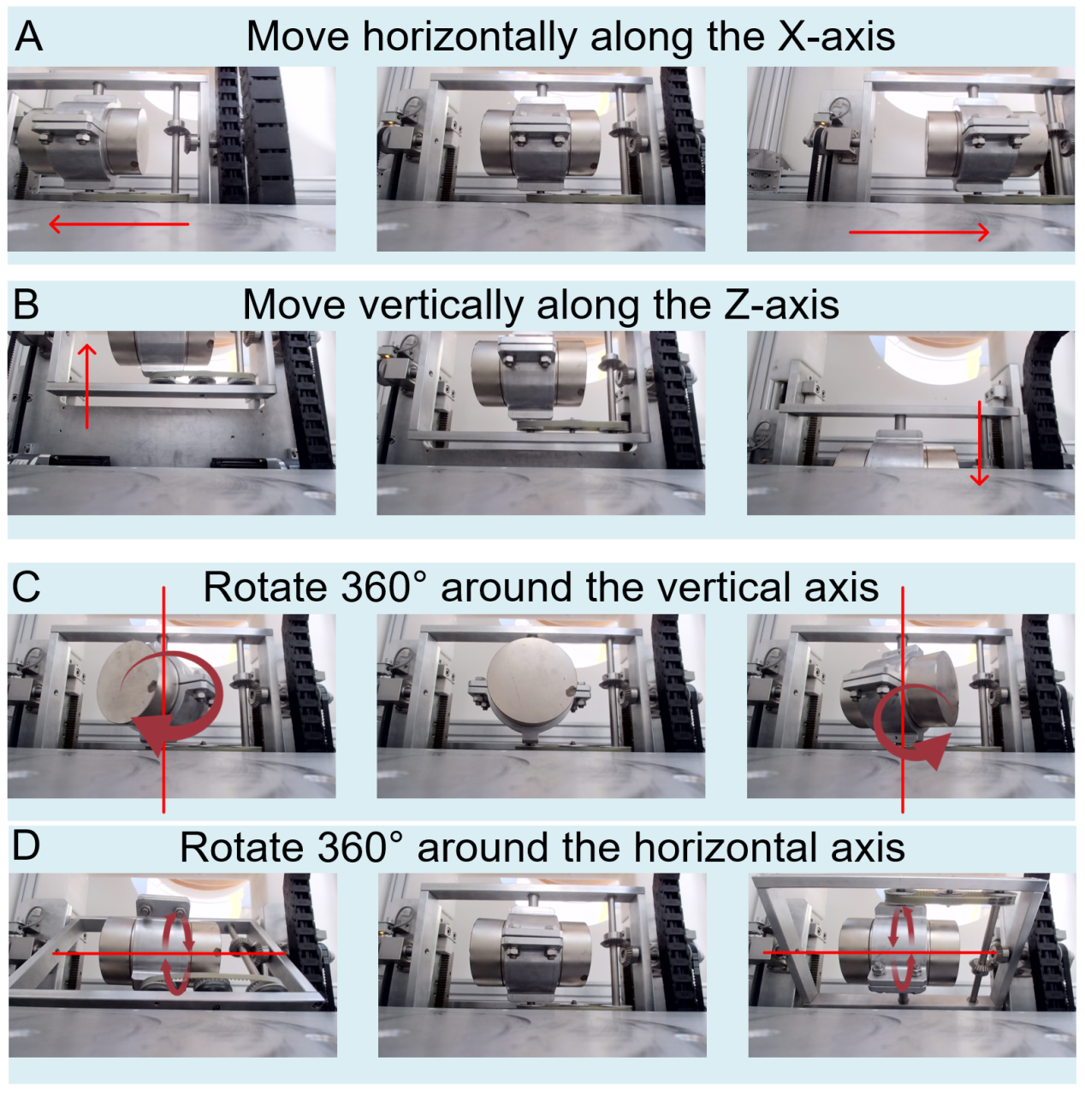


Figure S18. (A) Horizontal movement of the large permanent magnet along the X-axis. (B) Vertical movement of the large permanent magnet along the Z-axis. (C) 360° rotation of the large permanent magnet around the vertical axis. (D) 360° rotation of the large permanent magnet around the horizontal axis.


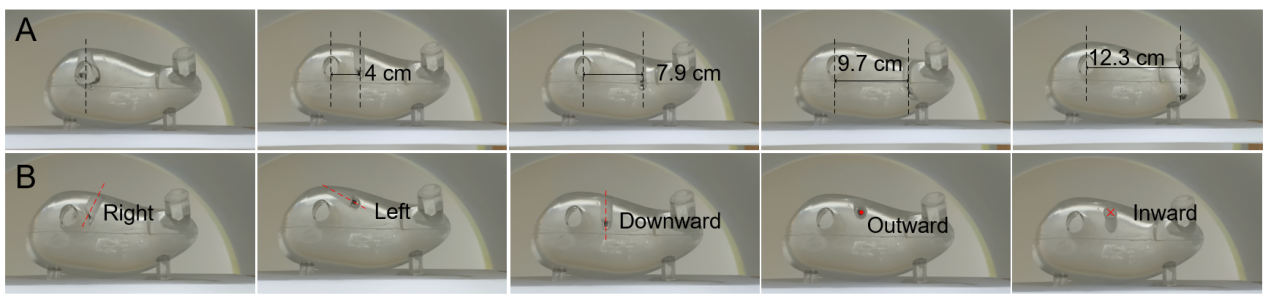


Figure S19. (A) Translational movement of NIR‑II FICE along the inferior wall of the gastric model.​ (B) Rotational maneuvers of NIR‑II FICE at the inferior wall of the gastric model: rightward, leftward, vertically downward, outward, and inward rotations.​


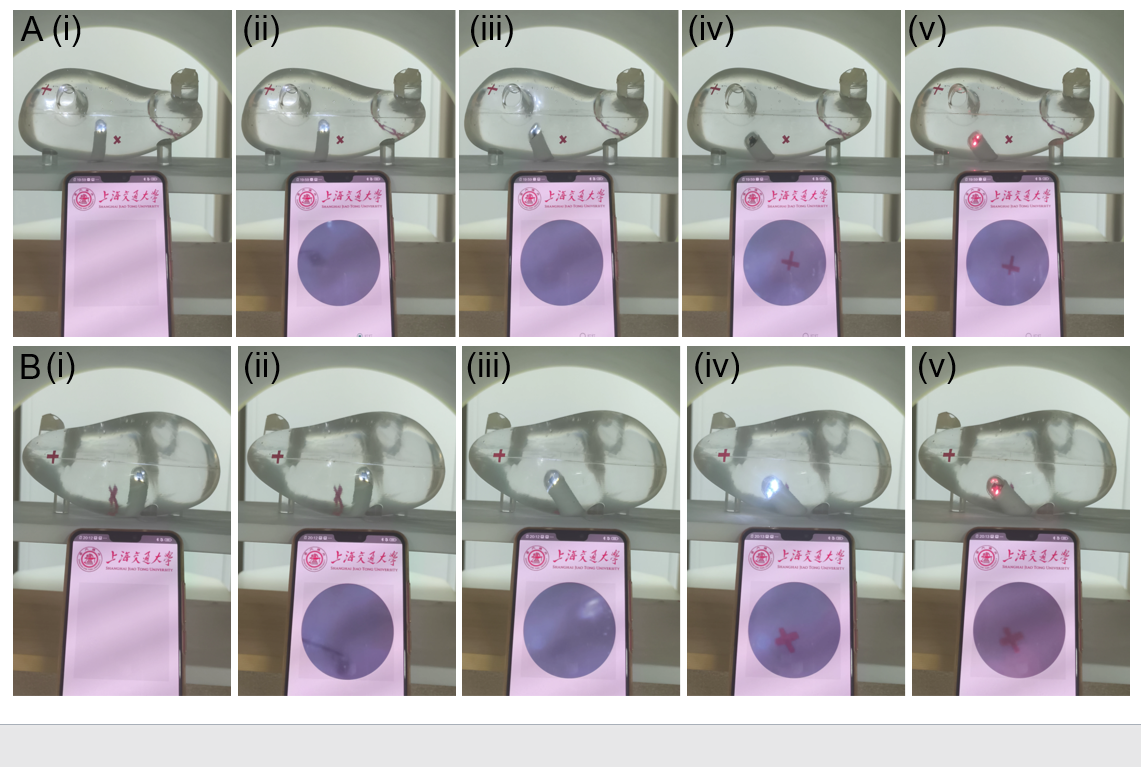


Figure S20. (A) (i) Experimental setup for the motion, positioning, image acquisition, and data transmission of the NIR-II FICE under the synergistic operation of wireless power supply and magnetic control devices at marked lesion location 2, (ii) image receiving equipment connection, (iii) motion and positioning of the NIR-II FICE, (iv) image acquisition and wireless transmission under white-light imaging, and (v) image acquisition and wireless transmission under fluorescence imaging. (B) (i) Experimental setup for the motion, positioning, image acquisition, and data transmission of the NIR-II FICE under the synergistic operation of wireless power supply and magnetic control devices at marked lesion location 3, (ii) image receiving equipment connection, (iii) motion and positioning of the NIR-II FICE, (iv) image acquisition and wireless transmission under white-light imaging, and (v) image acquisition and wireless transmission under fluorescence imaging.


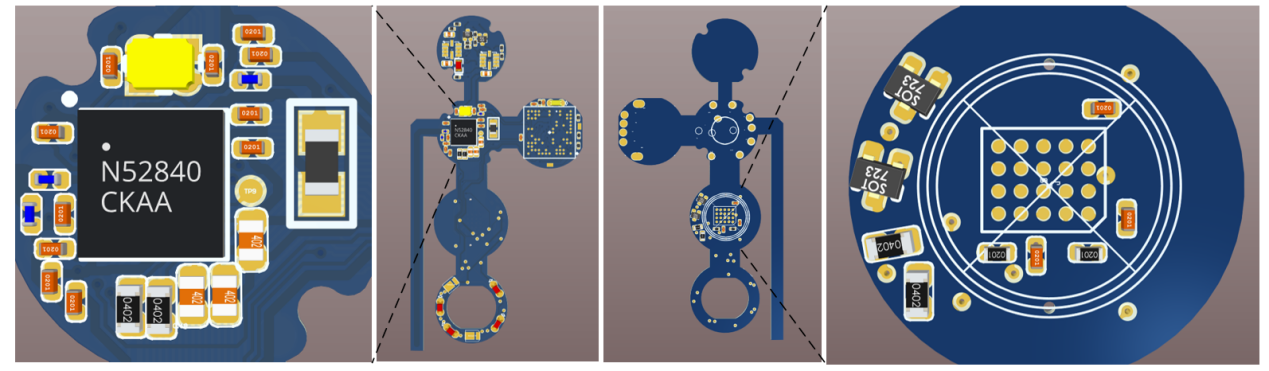


Figure S21. Encapsulation design of the capsule endoscope.


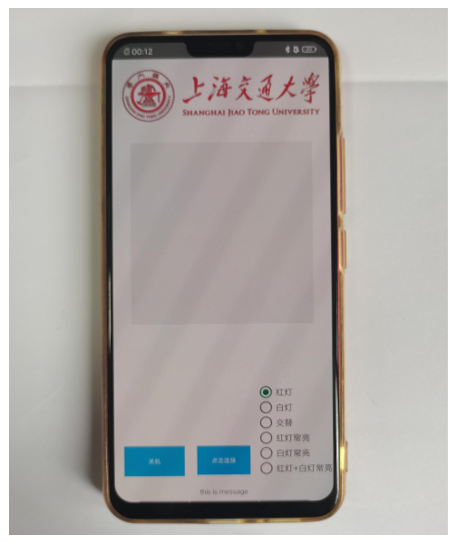


Figure S22. Image receiver operation interface.


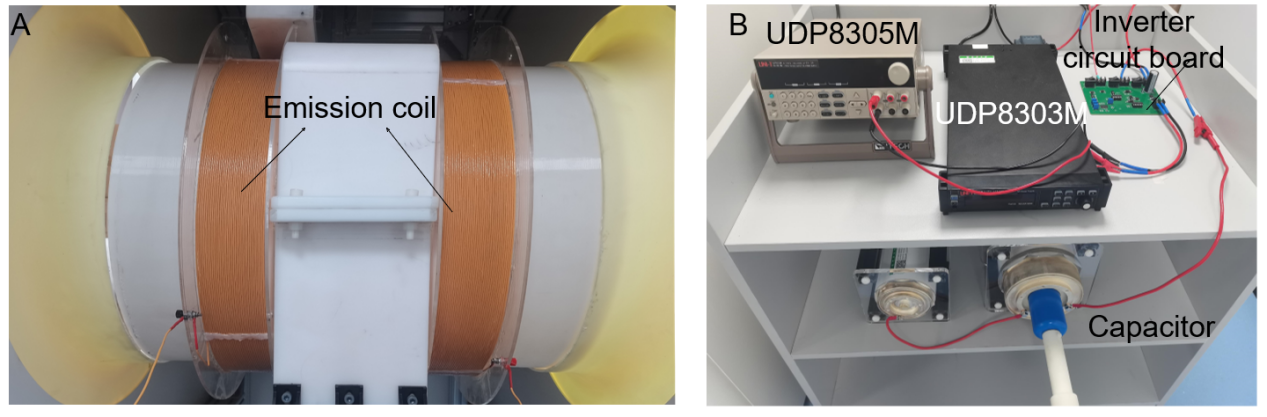


Figure S23. (A)Wireless power supply transmitting coil. (B) Wireless energy supply driving device.


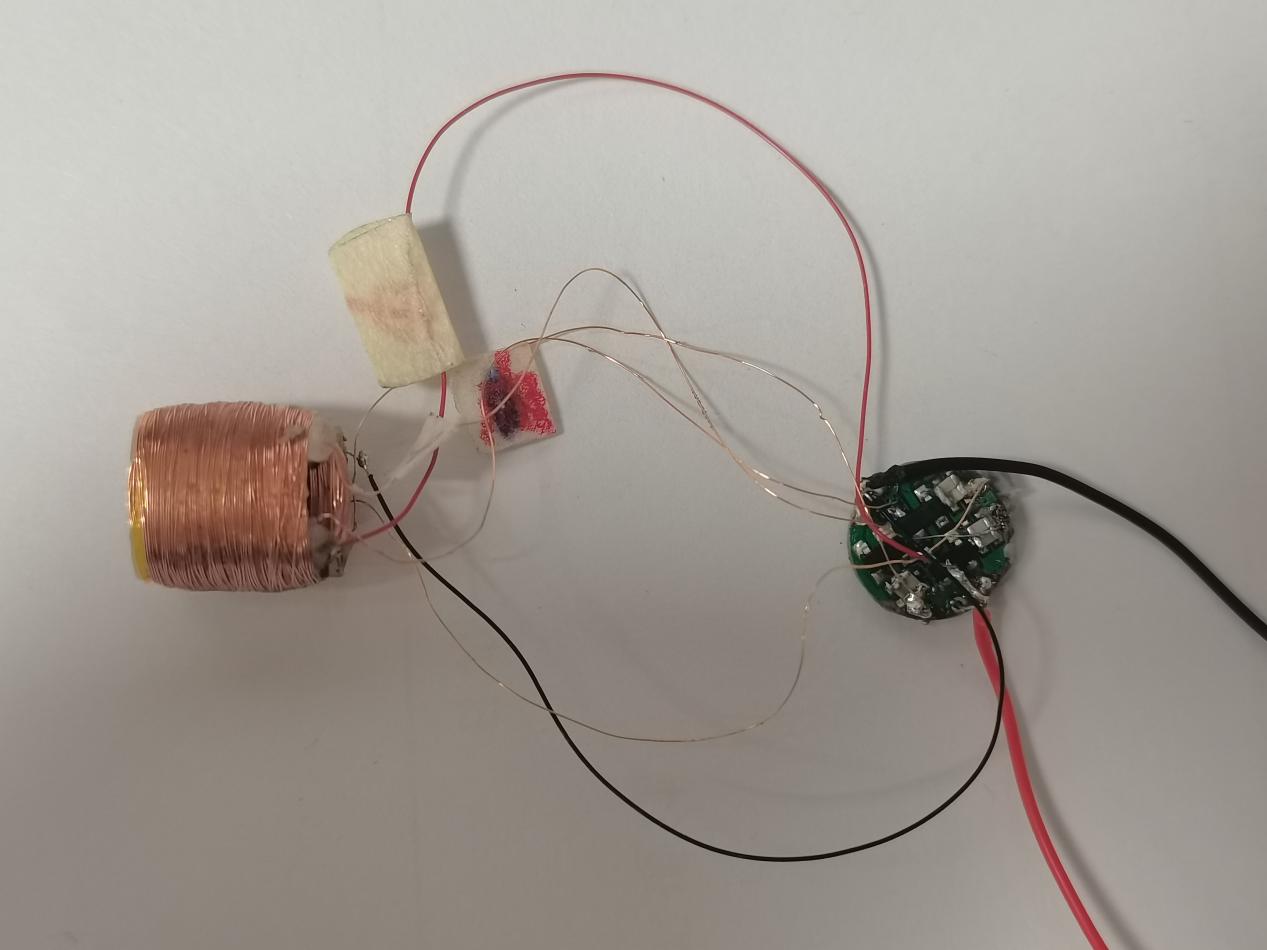


Figure S24. Wireless power supply receiving part.


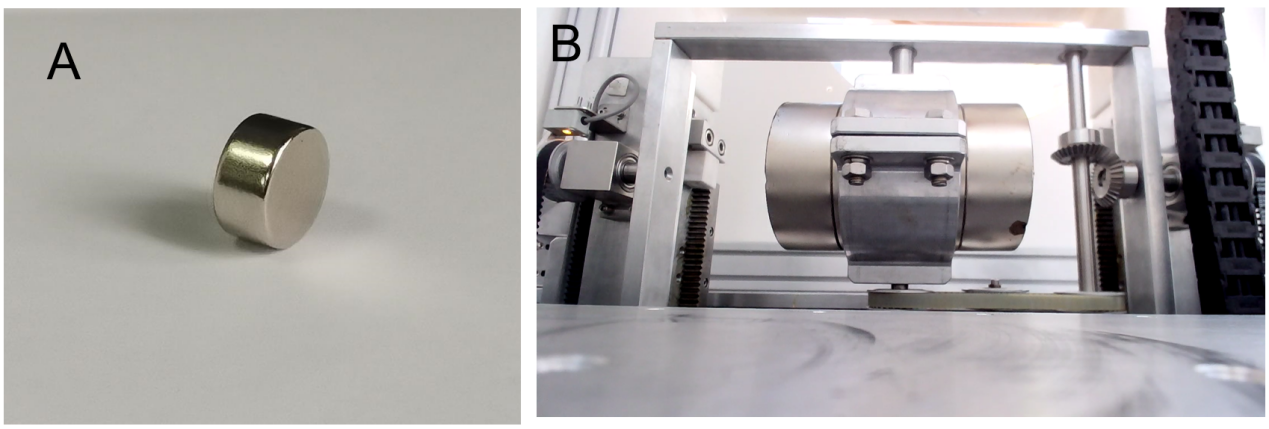


Figure S25. (A) Internal permanent magnet. (B) External permanent magnet.


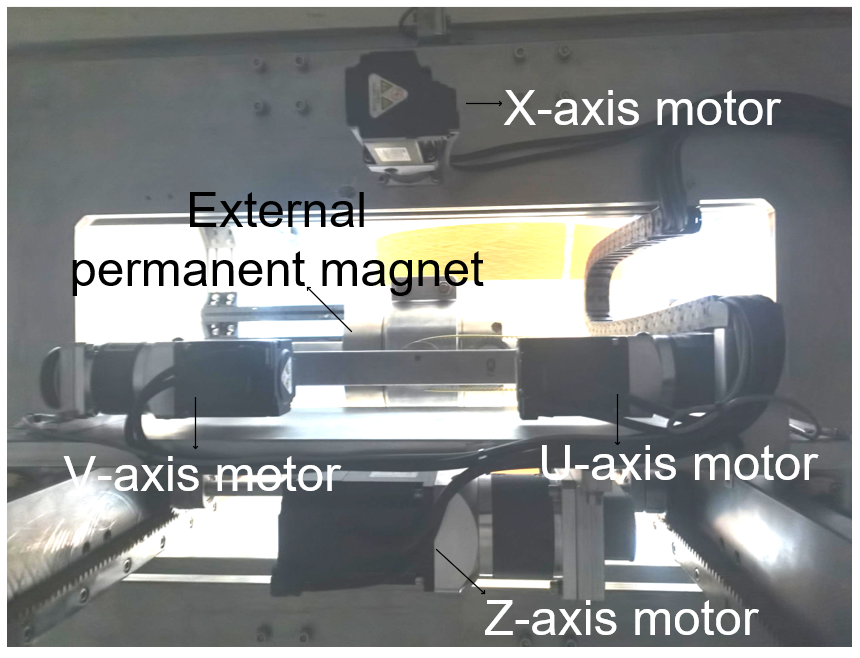


Figure S26. Mechanical structure for controlling external permanent magnets.


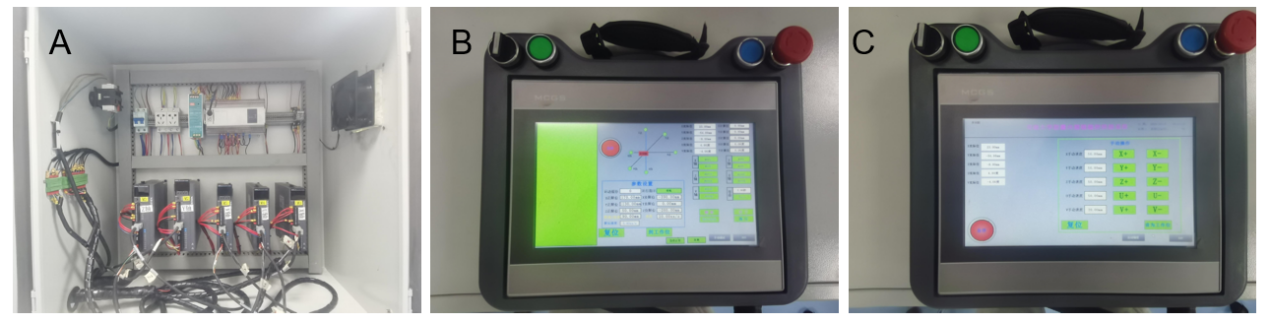


Figure S27. (A) Electric control cabinet for controlling external permanent magnets. (B) Control panel programming mode. (C) Manual mode of the control panel.


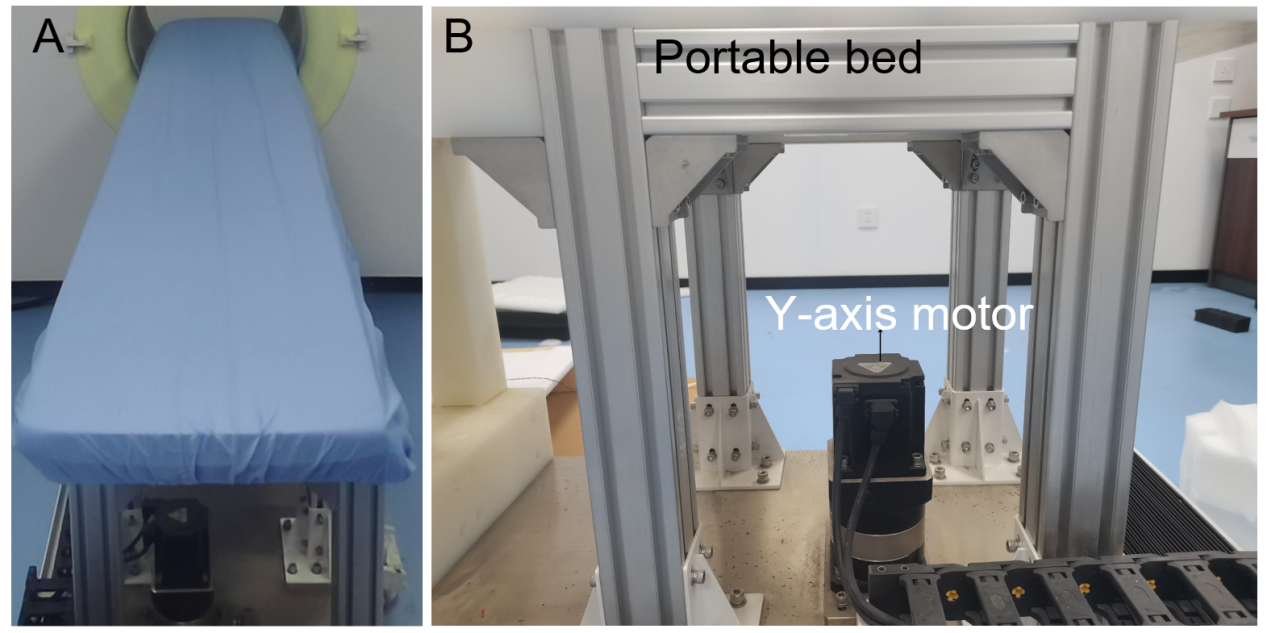


Figure S28. (A) Movable examination bed. (B) Control for the motor.


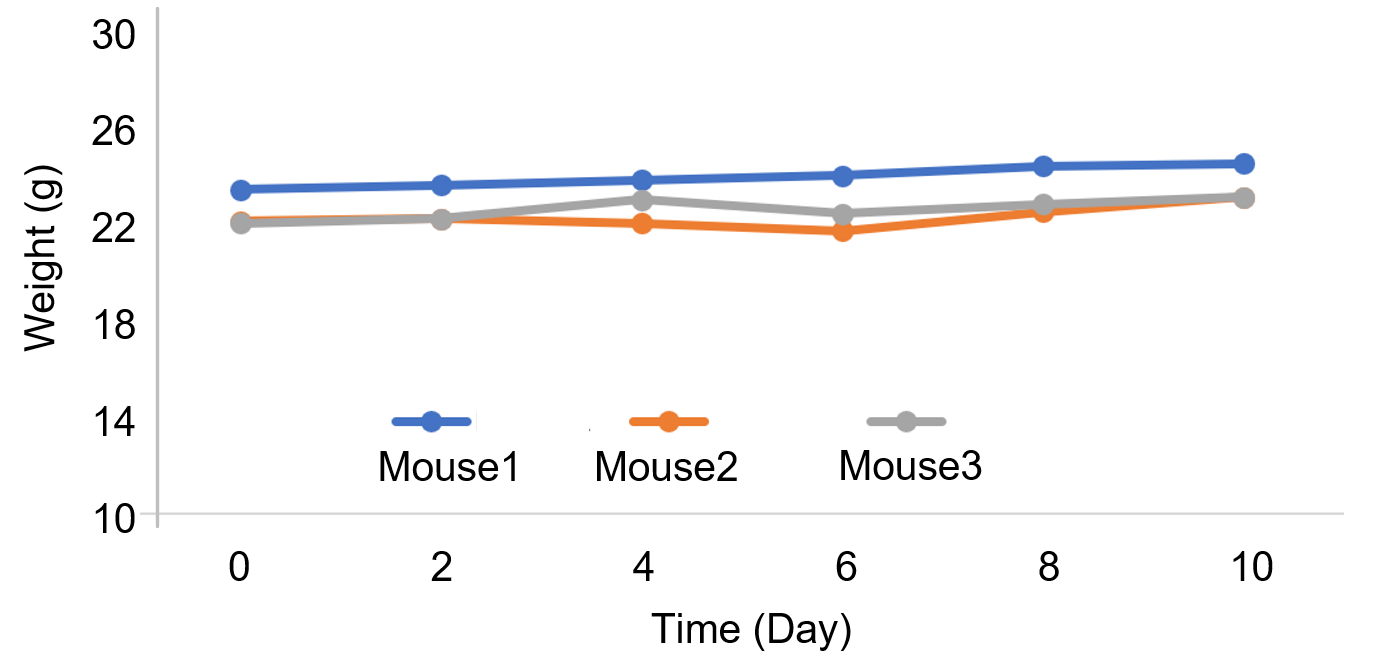


Figure S29. The weight change curve of tumor-bearing nude mice within ten days.


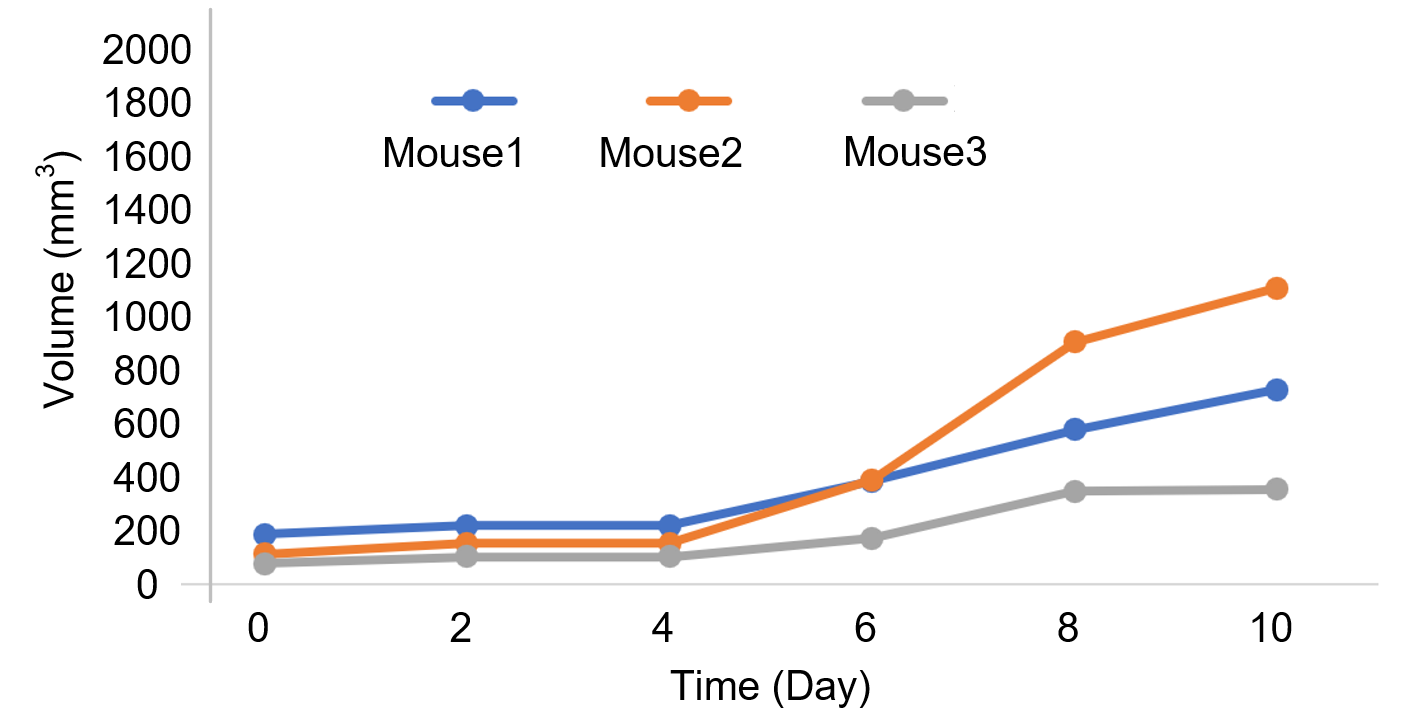


Figure S30. Growth curves of tumor volume in nude mice over a 10-day period.

Table S1. The weight of the mouse (g)

|  | Day 0 | Day 2 | Day 4 | Day 6 | Day 8 | Day 10 |
| --- | --- | --- | --- | --- | --- | --- |
| Mouse 1 | 23.5 | 23.7 | 23.9 | 24.1 | 24.5 | 25.1 |
| Mouse 2 | 22.2 | 22.3 | 22.1 | 21.8 | 22.6 | 23.2 |
| Mouse 3 | 22.1 | 22.3 | 23.1 | 22.5 | 22.9 | 23.2 |

Table S2. The volume of the tumor (mm^3^)

|  | Day 0 | Day 2 | Day 4 | Day 6 | Day 8 | Day 10 |
| --- | --- | --- | --- | --- | --- | --- |
| Mouse 1 | 190.56 | 223.21 | 223.21 | 390.6195 | 582.824 | 733.862 |
| Mouse 2 | 116.30 | 156.15 | 156.156 | 394.075 | 912.99 | 1115.609 |
| Mouse 3 | 80.35 | 105.72 | 105.72 | 174.945 | 351.68 | 358.45 |
